# Investigating Contextual Factors Affecting Digital Transformation Synergy and Supply Chain Financial Sustainability Models

**DOI:** 10.12688/f1000research.174111.1

**Published:** 2026-02-12

**Authors:** Nguyen Thi Hang, Nam Vu Quynh

**Affiliations:** 1 Thai Nguyen University of Information and Communication Technology, Thai Nguyen, Vietnam; 2 Thai Nguyen University of Economics and Business Administration, Thái Nguyên, Thai Nguyen, Vietnam

**Keywords:** Digital transformation, Supply chain finance, Sustainable financial model, Strategic integration, Supply chain adaptability

## Abstract

**Background:**

In the context of accelerating digital transformation, the alignment between digital transformation initiatives and sustainable supply chain finance has become an important issue for firms seeking to improve operational coordination and financial efficiency. This alignment, however, is influenced not only by technological adoption but also by organizational and contextual conditions such as governance structures, strategic orientation, and resource availability. Existing studies have not sufficiently examined how these contextual factors jointly shape the integration of digital transformation and supply chain finance.

**Methods:**

This study adopts a mixed-methods approach that combines quantitative analysis based on survey data with qualitative insights. A multidimensional analytical framework is developed to examine the contextual factors affecting the integration between digital transformation and sustainable supply chain finance. The framework includes leadership vision, strategic orientation, information-sharing mechanisms, supplier collaboration, organizational resources, process readiness, and access to financial and credit resources.

**Results:**

The findings indicate that these contextual factors collectively influence the degree of alignment between digital transformation initiatives and sustainable supply chain financial models. Leadership vision and clear strategic orientation play a central role in guiding coordination among supply chain actors. Effective information-sharing mechanisms support collaboration and transparency across partners. In addition, organizational resources and process readiness significantly affect the feasibility and effectiveness of implementing digital solutions linked to supply chain finance. Access to financial and credit resources also conditions firms’ ability to operationalize such initiatives.

**Conclusion:**

The study provides empirical evidence on how organizational and contextual conditions shape the integration of digital transformation with sustainable supply chain finance. The results suggest that alignment depends on a combination of strategic direction, internal capabilities, and coordination mechanisms, offering useful implications for firms seeking to design digital transformation initiatives that are consistent with their organizational capacities and operational contexts.

## 1. Introduction

In the context of globalization and the rapid evolution of the digital economy, sychronizing digital transformation (DT) and sustainable financial models in supply chains (SCF) has become an urgent requirement for enhancing enterprise competitiveness and adaptability. The Fourth Industrial Revolution with the emergence of advanced technologies such as artificial intelligence, blockchain and the Internet of Things (IoT), has reshaped traditional supply chain operations, necessitating a shift toward digital ecosystems. ^5^ Simultaneously, the financial sustainability of supply chains including financing mechanisms, credit support and capital optimization has become a key concern amid economic uncertainty and rising operational costs. ^7^ This study focuses on analyzing the contextual factors influencing the synchronization between digital transformation and sustainable supply chain finance models, arguing that this convergence is a core driver for innovation, economic viability and market responsiveness.

The concept of digital transformation in supply chain management refers to the strategic integration of digital technologies to optimize processes, enhance visibility and improve decision-making across the entire supply network. ^11, 12, 14, 28– 35^ Recent studies suggest that digital transformation enables real-time data sharing, predictive analytics and process automation, thereby minimizing waste and enhancing supply chain agility. ^6, 21– 27^ However, the success of digital transformation depends on a set of multidimensional contextual factors, including leadership vision, organizational readiness and inter-organizational collaboration. Likewise, sustainable supply chain finance involves deploying financial tools such as trade finance, supply chain loans and credit guarantees to ensure liquidity and long-term operational stability. The convergence of these two domains is particularly critical in emerging economies, where resource and infrastructure constraints pose significant barriers to adoption. ^1^

In the Vietnamese context, the northern mountainous provinces represent a unique case study, with an emerging entrepreneurial ecosystem and increasing emphasis on sustainable development. ^36^ This study emphasizes the need to explore how contextual factors shape the integration between digital transformation and supply chain finance, especially in resource scarce regions.

The proposed research model identifies eight key contextual factors influencing this integration: (1) TN – Leadership vision and commitment to implementing digital transformation and supply chain finance; (2) DH – Strategic orientation toward sustainable supply chain management; (3) XD – Information exchange mechanisms within the supply chain; (4) HTPT – Collaborative development of sustainable strategic relationships with suppliers; (5) NLDN – Organizational resources affecting digital transformation and financial optimization; (6) SSDN – Readiness in supply, production, logistics and financial management processes; (7) CCTC – Provision of financial and credit support across the supply chain; (8) CDSTTCCU – Behaviors related to digital transformation and supply chain finance. These elements form a multidimensional framework that illuminates the interaction between technological innovation and financial sustainability.

Existing literature highlights the transformative potential of digital transformation in supply chain management. For example, emphasizes the role of digital tools in improving supply chain visibility, ^13^ while under scores the importance of blockchain in securing financial transactions. ^20^ However, the literature also reveals gaps in understanding how contextual factors mediate this transformation, especially in developing economies. Suggest that leadership commitment and resource availability are critical drivers, yet the specific role of supplier collaboration and process readiness remains under-explored. ^21^ Moreover, the financial aspect of supply chain finance, as discussed by Hofmann et al. (2011), demands a nuanced approach when integrated with digital transformation an area lacking empirical evidence. ^15– 17^

This study addresses these gaps by employing a mixed-methods approach, combining quantitative survey data analysis with qualitative insights from case studies in Northern Vietnam. The study contends that synchronizing digital transformation and supply chain finance is not merely a technical endeavor but a strategic alignment influenced by context-specific economic variables. For instance, limited technological infrastructure in mountainous provinces necessitates innovative financial models to support digital adoption a challenge this research seeks to clarify. By examining the proposed factors, the study aims to provide a comprehensive understanding of how businesses can leverage digital transformation to enhance the sustainability of supply chain finance, thereby contributing to broader discourse on digital economic transformation.

The significance of this study lies in its potential to inform both policy and managerial practice. In Vietnam, where the government has launched a National Digital Transformation Program through 2025 with a vision toward 2030, the findings may guide policymakers in designing incentives to integrate digital transformation and supply chain finance. For businesses, the research offers practical insights into optimizing resource allocation and fostering collaborative networks. Moreover, the academic contribution is substantial, as it expands the theoretical framework of supply chain management by integrating digital transformation and financial sus tainability through a contextual lens an area ripe for deeper scholarly exploration.

## 2. Literature review

The synchronization between digital transformation (DT) and sustainable supply chain finance (SCF) has become a key focus area in the era of globalization and Industry 4.0. ^2^ Contextual factors governing this integration revolve around critical elements such as leadership vision (TN), strategic orientation (DH), information exchange mechanisms (XD), supplier collaboration (HTPT), organizational resources (NLDN), process readiness (SSDN), financial and credit support (CCTC) and digital transformation and supply chain finance behavior (CDSTTCCU). These factors are examined in the context of a developing country Vietnam particularly in the northern mountainous provinces, where limited resources and infrastructure demand innovative solutions adapted to local realities.

Digital transformation in supply chain management (SCM) has been widely studied as a means to optimize processes and enhance efficiency. Campos al. (2016) emphasize that digital tools such as predictive analytics and blockchain improve supply chain visibility and automation, reducing waste and enhancing flexibility. ^9, 10^ However, argues that the success of digital transformation depends on contextual elements such as leadership vision (TN) and organizational resources (NLDN). ^18^ The work of Lerman et al. (2022) also underscores that leadership commitment is a key driver of digital adoption, particularly in emerging economies where technological infrastructure is limited. ^19, 20^

Supply chain finance, as defined by Hofmann and Belin (2011), is a financial mechanism that leverages the creditworthiness of large firms to support liquidity for smaller suppliers, especially small and medium-sized enterprises (SMEs). ^8, 17^ Tools such as accounts receivable financing and invoice discounting similar to the solutions provided by Techcombank help ease cash flow pressures and improve working capital ratios. Nevertheless, Gelsomino et al. (2016) note that the integration between SCF and DT requires effective information sharing mechanisms (XD) and strategic collaboration with suppliers (HTPT). These studies highlight that process readiness (SSDN) and financial credit support (CCTC) are crucial for ensuring financial sustainability in supply chains. ^3, 4^

Although existing literature provides a solid theoretical foundation, significant gaps remain in understanding how contextual factors interact in developing economies. The interaction between factors such as credit support (CCTC), strategic orientation (DH), process readiness (SSDN) and digital transformation and SCF behavior (CDSTTCCU) represents a core dynamic for integrating DT and SCF in such contexts. These factors not only shape financial structures but also influence the adaptability of supply chains. However, knowledge of how these variables interact is still limited particularly when SMEs face unique challenges. This points to the need for deeper investigation into the complex interplay of these factors to develop contextually appropriate solutions.

According to the 2024 report by the IFC and WTO, SMEs account for approximately 40% of GDP and 60% of employment in Vietnam, yet only 20% of them have access to bank financing. This challenge highlights the vital role of CCTC in supporting liquidity and encouraging SME participation in global supply chains. Still, current studies do not fully explore how factors such as DH and SSDN moderate the integration between DT and SCF, especially in overcoming financial barriers. This creates a significant research gap, requiring empirical analysis to propose innovative financing models.

Moreover, there remains a shortage of empirical research on CDSTTCCU in resource-scarce regions such as northern mountainous Vietnam. This area, characterized by limited digital infrastructure and high operating costs, represents a unique context in which SMEs face dual challenges: constrained financial resources and limited access to digital technologies. The lack of empirical data on DT and SCF adoption behavior here hinders the development of appropriate strategies. For example, while the implementation of digital platforms such as blockchain could enhance financial transparency, such efforts may be impeded by low process readiness (SSDN) a relationship yet to be thoroughly explored. This research gap calls for localized studies to better understand how contextual factors interact in specific environments.

This gap is significant both theoretically and practically. Theoretically, the lack of understanding regarding contextual interactions limits the development of comprehensive DT-SCF integration models. Practically, it impacts the formulation of support policies for SMEs especially within the scope of Vietnam’s National Digital Transformation Program to 2025. By focusing on factors such as TN, DH, XD, HTPT, NLDN, SSDN, CCTC and CDSTTCCU, this paper seeks to bridge the gap by providing empirical data from the northern mountainous region, thereby informing strategies to enhance economic competitiveness. By addressing these gaps, the study contributes both practical and theoretical solutions to foster sustainable supply chain development in the digital economy.

**Table 1.** Contextual factors influencing the synchronization of digital transformation and SCF.

Factor	Description	References
TN	Vision and commitment of company leadership toward implementing the integration of digital transformation and supply chain finance.	Dharmayanti et al., 2023; Alkaraan et al., 2022
DH	Strategic orientation and deployment of sustainable supply chain management strategies by the enterprise.	Deckert, 2023; Campos et al., 2016; Cherrafi et al., 2018
XD	Establishing effective mechanisms for information exchange, transmission and reception throughout the entire supply chain.	Gelsomino et al., 2016; Guandalini, 2022; Mariadoss, 2011
HTPT	Collaboration and development of sustainable strategic relationships between enterprises and their suppliers.	Hofmann & Belin, 2011; He, 2020; Omrani, 2022
NLDN	Organizational resource factors influencing the implementation of digital transformation and optimization of supply chain finance.	Ali, 2023; Jia, 2025; Nejati, 2017; Neri, 2023; Sullivan et al., 2018
SSDN	The enterprise’s readiness across supply, production, processing, transportation, capital management, contracts and order fulfillment processes, impacting the implementation of digital transformation and the optimization of supply chain finance.	Caniato et al., 2019; Plekhanov et al., 2023; Zhao et al., 2024; Sun et al., 2025
CCTC	Provision of financial resources and credit support across the entire supply, production, processing, transportation, capital management, contracts and order fulfillment processes, influencing the implementation of digital transformation and the optimization of supply chain finance.	Wang et al., 2024; Zangiacomi et al., 2020
CDSTTCCU	Behavior related to the implementation of digital transformation and supply chain finance.	Weritz et al., 2025; Yuan et al., 2024

## 3. Research model and hypotheses

### 3.1 Research model

In the context of the growing influence of digital transformation (DT) on supply chain operations, the development of a sustainable supply chain finance (SCF) model must be aligned with the internal contextual factors of enterprises. This research model is constructed based on an integrated theoretical foundation, drawing from supply chain management theory, the Resource-Based View (RBV) proposed by Wernerfelt (1984) following Penrose’s (1959) earlier work and recent studies on digital transformation in logistics and supply chain finance.

The objective of the model is to identify contextual factors influencing digital transformation and supply chain finance behavior (CDSTTCCU), thereby elucidating the role of synchronization between digital transformation and enterprises’ sustainable financial models. Accordingly, the research proposes a model consisting of seven independent variables and one dependent variable:

Independent Variables:

Vision and commitment of company leadership (TN): This foundational factor reflects the degree of commitment, strategic orientation and leadership capability in driving digital transformation and sustainable development in the supply chain. According to RBV, leadership plays a pivotal role in shaping internal competitive advantage.

Strategic orientation for sustainable supply chain management (DH): This indicates the enterprise’s capability in formulating policies and development plans for the supply chain based on economic, social and environmental sustainability criteria.

Mechanisms for information sharing, transmission and reception (XD): Effective information sharing within and across partner organizations is a prerequisite for implementing digital technologies and managing financial flows in the supply chain.

Collaboration and development of strategic supplier relationships (HTPT): The level of trust, commitment and long-term interaction between the enterprise and its suppliers facilitates the sharing of financial data and improves the efficiency of SCF mechanisms.

Enterprise resources (NLDN): This includes financial, human, technological, managerial and infrastructural resources core capabilities essential for digital transformation and sustainable SCF model implementation.

Enterprise readiness (SSDN): Reflects the organizational, procedural and personnel preparedness for synchronously deploying digitalization across production, processing, logistics and contract management.

Provision of financial and credit support within the supply chain (CCTC): A critical factor to ensure liquidity and efficient cash flow circulation, maintaining the stability and sustainability of the entire supply chain.

Dependent Variable:

Behavior of digital transformation and supply chain finance (CDSTTCCU): A synthesized outcome of the aforementioned factors, reflecting the extent to which digital technologies are applied in operations, financial management and the development of flexible, transparent and sustainable supply chain relationships.

### 3.2 Research hypotheses

Based on the proposed model, the following hypotheses are developed:

H1: Vision and commitment of company leadership (TN) positively influence CDSTTCCU behavior.

H2: Strategic orientation for sustainable supply chain management (DH) positively influences CDSTTCCU behavior.

H3: Mechanisms for information sharing and reception (XD) positively influence CDSTTCCU behavior.

H4: Level of collaboration with suppliers (HTPT) positively influences CDSTTCCU behavior.

H5: Enterprise resources (NLDN) positively influence CDSTTCCU behavior.

H6: Enterprise readiness (SSDN) positively influences CDSTTCCU behavior.

H7: Provision of financial and credit support in the supply chain (CCTC) positively influences CDSTTCCU behavior.

## 4. Research methodology

This study employs a mixed-methods approach that integrates both quantitative and qualitative techniques to comprehensively evaluate contextual factors affecting the synchronization of digital transformation and sustainable financial models within supply chains. The methodology is grounded in a multidimensional theoretical framework, combining the Resource-Based View (RBV) and Dynamic Capability Theory to explore relationships among the key variables identified in the research.

This study involved human participants. Before data collection, all participants were clearly informed about the purpose of the study, the voluntary nature of their participation, and their right to withdraw at any time without consequences. Informed consent was obtained voluntarily from all participants before they took part in the survey. The survey participants were all senior executives and managers within the company, excluding minors. Informed consent was obtained verbally, as data was collected via questionnaires. This approach was deemed appropriate to ensure convenience and confidentiality for participants. No minors participated in this study.

Data collection was conducted in two phases. The first phase involved a qualita-tive study through semi-structured interviews with 25 senior executives from en-terprises and banks in Vietnam. These participants were selected based on their experience in digital transformation and supply chain finance strategies. The interviews focused on gathering insights related to leadership vision (TN), strategic orientation (DH), mechanisms for information exchange (XD), supplier collaboration (HTPT), enterprise resources (NLDN), organizational readiness (SSDN) and the provision of financial and credit support across the supply chain (CCTC).

The second phase involved a quantitative study using a structured survey distributed to 78 representative companies, including both small and medium-sized enterprises (SMEs) and large corporations operating within Vietnam’s supply chain sector. A five-point Likert scale was employed to assess perceived impacts of the identified factors on the convergence between digital transformation and supply chain finance (CDSTTCCU). A stratified random sampling method was applied to ensure representativeness across industry types and firm sizes. The response rate reached 84.6%, yielding 66 valid responses and forming a reliable dataset for statistical analysis.

The data analysis procedures were designed to assess the model’s adequacy and reliability using linear regression techniques. Initially, the study calculated the R ^2^ (R Square) and Adjusted R ^2^ values to evaluate the explanatory power of the model for the dependent variable. This step was crucial for determining the overall model fit. Subsequently, model significance was tested using the Sig. value of the F-test from the ANOVA table, with a significance threshold set at p < 0.05 to confirm model appropriateness with the empirical data.

To ensure the stability of the model, multicollinearity was examined. This included analyzing the statistical significance of independent variables via the “Sig.” column in the regression output to determine which variables had significant effects (p < 0.05). Additionally, the Variance Inflation Factor (VIF) from the Collinearity Statistics was used to detect multicollinearity, with an acceptable threshold of VIF < 10.

Finally, the regression assumptions were validated through three visual diagnostic tools: the normalized residual histogram to assess the normal distribution of residuals; the Normal P-P Plot to evaluate the fit with the normal distribution; and the scatter plot to test the linear relationship assumption between independent and dependent variables. All analyses were performed using SPSS software to ensure methodological rigor and scientific accuracy in the findings.

## 5. Results

### 5.1 Scale reliability testing

The results of the scale reliability test indicate a high level of internal consistency. The Cronbach’s Alpha coefficients of the measurement scales range from 0.766 to 0.960, all exceeding the acceptable threshold of 0.6. Additionally, all 49 observed variables have item-total correlation coefficients greater than 0.3, confirming their suitability for further factor analysis (see Table 2).

**Table 2.** Cronbach’s alpha reliability coefficients.

Measurement scale	Encryption	Cronbach’s alpha	Number of observed variables
**Vision and commitment of company leadership toward implementing the integration of digital transformation and supply chain finance.**	TN	0,958	6
**Strategic orientation and deployment of sustainable supply chain management strategies by the enterprise.**	DH	0,786	6
**Establishing effective mechanisms for information exchange, transmission and reception throughout the entire supply chain.**	XD	0,960	7
**Collaboration and development of sustainable strategic relationships between enterprises and their suppliers.**	HTPT	0,904	7
**Organizational resource factors influencing the implementation of digital transformation and optimization of supply chain finance.**	NLDN	0,953	12
**The enterprise’s readiness across supply, production, processing, transportation, capital management, contracts and order fulfillment processes, impacting the implementation of digital transformation and the optimization of supply chain finance.**	SSDN	0,945	4
**Provision of financial resources and credit support across the entire supply, production, processing, transportation, capital management, contracts and order fulfillment processes, influencing the implementation of digital transformation and the optimization of supply chain finance.**	CCTC	0,887	4
**Behavior related to the implementation of digital transformation and supply chain finance.**	CDSTTCCU	0,766	3

(Source: SPSS analysis results).

The scale reliability test results in this study indicate high internal consistency, with Cronbach’s Alpha coefficients ranging from 0.766 to 0.960, all exceeding the minimum threshold of 0.6. This demonstrates strong reliability and internal consistency of the measurement scales ( Table 1), thereby confirming that the observed variables accurately reflect their corresponding theoretical constructs. Notably, the “Information Exchange Mechanism” (XD) scale achieved the highest Alpha value of 0.960, followed closely by the “Leadership Vision” (TN) scale at 0.958, indicating excellent reliability. On the other hand, the “Behavior of Digital Transformation and Supply Chain Finance Implementation” (CDSTTCCU) scale reported the lowest Alpha value at 0.766, which still meets acceptable research standards.

All 49 observed variables recorded Corrected Item-Total Correlation values greater than 0.3 ( Table 2), satisfying the prerequisite for conducting Exploratory Factor Analysis (EFA). This suggests that each item contributes positively to the overall construct, with no item requiring elimination, thus maintaining the integrity of the measurement model. The number of observed variables per scale ranged from 3 (CDSTTCCU) to 12 (NLDN), reflecting varying levels of detail across scales. In particular, the “Enterprise Resources” (NLDN) scale, consisting of 12 items, indicates the complexity and multidimensionality of resource assessment.

These findings affirm the scientific rigor and data reliability, providing a solid foundation for subsequent statistical analyses such as EFA or regression modeling. Nonetheless, it is worth noting that scales with lower Alpha values (e.g., CDSTTCCU) may benefit from additional observed items in future research to enhance precision. Overall, the current scales are sufficiently robust to capture the relationship between contextual factors and the synchronization of digital transformation within the supply chain context in Vietnam.

### 5.2 Exploratory Factor Analysis (EFA)

*EFA for Independent Variables*

Kaiser-Meyer-Olkin Measure of Sampling Adequacy (KMO) = 0.587.

The results of the factor analysis indicate that the KMO value is 0.587, which exceeds the minimum threshold of 0.5, confirming the suitability of the dataset for factor analysis. The Bartlett’s Test of Sphericity yields a Chi-square value of 3802.120 with a significance level (Sig.) of 0.000, which is less than 0.05. This result suggests that the observed variables are significantly correlated with each other and the factor analysis is statistically meaningful (see Table 3). Consequently, the null hypothesis (H0), stating that the variables are uncorrelated in the population, is rejected. This implies that the correlation matrix is not an identity matrix and the data meets the necessary conditions for factor extraction.

**Table 3.** KMO and Bartlett’s test.

Kaiser-Meyer-Olkin Measure of Sampling Adequacy		.587
Bartlett’s Test of Sphericity	Approx. Chi-Square	3802.120
	df	1035
	Sig.	.000

(Source: SPSS analysis results)

The factor extraction was conducted using Principal Component Analysis (PCA) with Varimax rotation to optimize factor interpretability. The results show that out of the original 46 observed variables, 6 were excluded and the remaining were grouped into 7 factors. The cumulative variance explained is 78.362%, which is significantly higher than the standard threshold of 50%, indicating that the seven extracted components account for 78.362% of the total variance in the dataset.

Additionally, all extracted factors have eigenvalues greater than 1, with the lowest eigenvalue among them being 1.165, thus meeting the standard criterion for factor retention.

**Table 4.** Rotated component matrix using varimax rotation method.

	Component						
	1	2	3	4	5	6	7
NLDN1	.941						
NLDN10	.934						
NLDN3	.932						
NLDN2	.907						
NLDN4	.863						
NLDN6	.841						
NLDN5	.818						
NLDN9	.719						
NLDN7	.710						
NLDN8	.701						
XD2		.968					
XD1		.952					
XD3		.906					
XD4		.876					
XD6		.841					
XD5		.827					
XD7		.783					
TN4			.913				
TN5			.912				
TN3			.864				
TN1			.855				
TN6			.849				
TN2			.784				
HTPT1				.891			
HTPT2				.851			
HTPT3				.826			
HTPT6				.750			
HTPT7				.728			
HTPT5				.720			
HTPT4				.667			
SSDN2					.914		
SSDN4					.873		
SSDN3					.868		
SSDN1					.794		
CCTC4						.842	
CCTC3						.826	
CCTC1						.751	
CCTC2						.724	
DH2							.786
DH1							.693
DH3							.586
DH4							.552

Extraction Method: Principal Component Analysis.

Rotation Method: Varimax with Kaiser Normalization.

All factor loadings are greater than 0.5 and there are no cases in which a variable loads significantly onto more than one factor with similar loading values. This indicates that the extracted factors satisfy both convergent and discriminant validity in the exploratory factor analysis (EFA). Furthermore, there is no cross-loading or confusion between constructs, meaning that items intended to measure one factor do not inadvertently align with another. Therefore, after the factor analysis, all independent constructs remain unchanged none were added or removed.

The rotated component matrix in the EFA confirms the discriminant validity of the constructs, with all factor loadings exceeding 0.5, which is above the commonly accepted threshold (see Table 4). Consequently, all observed variables are retained for subsequent analyses.

*EFA for the Dependent Variable*

The dependent variable, “Digital Transformation and Supply Chain Finance Implementation Behavior” (CDSTTCCU), consists of three observed indicators: CDSTTCCU1, CDSTTCCU2 and CDSTTCCU3.

The results of the KMO and Bartlett’s Test, Total Variance Explained and Rotated Component Matrix are presented in Table 5 below.

**Table 5.** Results of KMO and Bartlett’s test.

Kaiser-Meyer-Olkin Measure of Sampling Adequacy	.656
Bartlett's Test of Sphericity Approx. Chi-Square	54.467
df	3
Sig.	.000

The results of the KMO and Bartlett’s Test indicate that the KMO coefficient reaches 0.656, satisfying the minimum threshold (> 0.6) required for factor analysis ( Table 5). Meanwhile, Bartlett’s Test of Sphericity yields a Chi-Square value of 54.467, with degrees of freedom (df ) = 3 and a significance level (Sig.) = 0.000 < 0.05. This confirms that the correlation matrix among the observed variables is statistically significant and distinct, thereby justifying the continuation of exploratory factor analysis (EFA). These findings demonstrate that the data are suitable and reliable for subsequent analyses within the proposed research model.

The results of the variance analysis indicate that the first factor has an eigenvalue of 2.049, accounting for 68.3% of the total variance surpassing the theoretical threshold of 50% as required by factor analysis principles ( Table 6). Since only one factor exhibits an eigenvalue greater than 1, the model satisfies the condition for principal component extraction, thereby ensuring theoretical generalizability. This confirms that all observed variables converge to measure a single latent construct, consistent with the theoretical framework concerning contextual factors influencing the synchronization of digital transformation and supply chain finance.

**Table 6.** Total variance explained.

Component	Initial Eigenvalues			Extraction sums of squared loadings		
	Total	% of variance	Cumulative %	Total	% of variance	Cumulative %
1	2.049	68.300	68.300	2.049	68.300	68.300
2	.617	20.570	88.870			
3	.334	11.130	100.000			

### 5.3 Analysis of R ^2^ (R Square) and Adjusted R ^2^

R Square represents the proportion of variance in the dependent variable that is explained by the independent variables in the regression model. A higher R Square value implies that the model explains a greater proportion of the variation in the dependent variable. However, R Square can be inflated by the number of independent variables included in the model. Therefore, Adjusted R Square is used to compensate for the potential overestimation, offering a more accurate measure by adjusting for the number of predictors that do not meaningfully contribute to the model. Both R Square and Adjusted R Square range from 0 to 1 ( Table 7).

**Table 7.** Model summary results.

Model summary ^b^					
Model	R	R square	Adjusted R square	Std. Error of the estimate	Durbin-Watson
1	.659 ^a^	.434	.365	.61939	1.734

^a^ Predictors: (Constant), TN, XD, HTPT, NLDN, SSDN, CCTC, DH.

^b^ Dependent Variable: CDSTTCCU.

The model demonstrates a moderate level of statistical significance, with an R ^2^ value of 43.4%. This indicates that approximately 43.4% of the variance in digital transformation and supply chain finance behavior is explained by contextual factors such as enterprise readiness (SSDN), information exchange mechanisms (XD), organizational resources (NLDN), leadership vision (TN) and strategic orientation (DH).

The Adjusted R ^2^ is about 7% lower than the R ^2^, suggesting that some of the independent variables included in the model may not contribute significantly or may introduce noise into the regression. Nonetheless, the gap is not substantial, implying that the model remains statistically reliable and applicable to real world contexts, particularly in the domain of organizational behavior management.

### 5.4 Testing the goodness of fit of the linear regression model

To assess the goodness of fit of the linear regression model with the observed data, it is necessary to conduct hypothesis testing based on the significance value (Sig.) of the F-statistic in the ANOVA table. This test determines whether the overall regression model provides a statistically significant fit to the data ( Table 8).

**Table 8.** ANOVA analysis results.

ANOVA ^a^						
Model		Sum of squares	df	Mean square	F	Sig.
1	Regression	17.040	7	2.434	6.345	.000 ^b^
	Residual	22.252	58	.384		
	Total	39.292	65			

^a^ Dependent Variable: CDSTTCCU.

^b^ Predictors: (Constant), TN, XD, HTPT, NLDN, SSDN, CCTC, DH.

The results indicate that the Total Sum of Squares (Total SS = 39.292) represents the overall variance of the dependent variable (CDSTTCCU). The variance explained by the regression model (Regression SS = 17.040) reflects the portion of variation in the dependent variable accounted for by the independent variables in the model (TN, XD, HTPT, NLDN, SSDN, CCTC, DH). The residual variance (Residual SS = 22.252) represents the portion of variation not explained by the model, attributable to random error.

F = 6.345: This statistic is computed by dividing the Mean Square of the regression by the Mean Square of the residuals. It is used to test the overall significance of the regression model. The corresponding p-value (Sig. = 0.000) is less than the conventional significance level of 0.05, leading to the rejection of the null hypothesis (H _0_) and acceptance of the alternative hypothesis (H _1_). This means that the regression model is statistically significant and fits the data.

Based on the ANOVA table, the significance level of the F-test is 0.000 < 0.05, confirming that the regression model is appropriate for explaining the relationship between the contextual factors and the dependent variable.

The regression model is statistically significant at the 5% level, indicating that at least one independent variable has a significant effect on the dependent variable, CDSTTCCU (Digital Transformation and Supply Chain Finance Implementation Behavior). This validates the model’s applicability for explaining or predicting behavioral outcomes based on the examined contextual factors.

The regression analysis further reveals that the model provides a moderate explanatory power. However, variables that lack statistical significance (such as HTPT and CCTC) may be excluded from the model through coefficient testing or stepwise regression techniques. Additionally, it is recommended to assess multicollinearity (using VIF) and check for homoscedasticity (constant variance of errors) to ensure the model’s robustness.

### 5.5 Multicollinearity diagnostics

To examine the presence of multicollinearity, the Variance Inflation Factor (VIF) was employed. Conventionally, a VIF value greater than 10 indicates significant multicollinearity among independent variables. However, for studies using 5-point Likert scales, researchers have recommended adopting a more conservative threshold of 2. Accordingly, if a variable exhibits a VIF of 2 or higher, multicollinearity is considered to exist. In such cases, the variable in question may not provide meaningful explanatory power regarding the variation of the dependent variable ( Table 9).

**Table 9.** Multicollinearity test results.

Coefficients ^a^								
Model		Unstandardized coefficients		Standardized coefficients	t	Sig.	Collinearity statistics	
		B	Std. Error	Beta			Tolerance	VIF
1	(Constant)	-.169	.753		-.225	.823		
	CCTC	-.163	.143	-.139	-1.142	.258	.659	1.518
	SSDN	.342	.123	.321	2.788	.007	.737	1.357
	NLDN	.252	.132	.215	1.910	.061	.769	1.301
	HTPT	-.064	.123	-.059	-.517	.607	.748	1.338
	XD	.278	.139	.236	1.998	.050	.700	1.428
	DH	.136	.180	.100	.757	.452	.564	1.772
	TN	.224	.135	.194	1.657	.103	.711	1.407

^a^ Dependent Variable: CDSTTCCU.

The regression analysis identified two contextual factors with statistically significant positive effects on the convergence of digital transformation and sustainable supply-chain finance:

Process Readiness(SSDN):β=0.342,p=0.007.

This was the strongest predictor, indicating that a firm’s readiness across its end-to-end supply, production, logistics, working-capital, contracting and order-management processes is a key driver of digital transformation and supply-chain finance integration.

Information−Sharing Mechanism(XD):β=0.278,p=0.050.

Effective channels for exchanging, transmitting and receiving information throughout the supply chain significantly enhance firms’ digital transformation behaviors.

All variance-inflation factors (VIFs) were below 2, confirming the absence of multicollinearity and indicating that the model is stable and reliable for practical application.

The standardized regression equation is therefore:

$$\text{CDSTTCCU}=0.321^{*}\text{SSDN}+0.236^{*}\mathrm{XD}+0.215^{*}\text{NLDN}+0.194^{*}\mathrm{TN}+0.100^{*}\mathrm{DH}–0.139^{*}\text{CCTC}–0.059^{*}\text{HTPT}$$

The regression results reveal that Process Readiness (SSDN) exhibits the highest standardized β coefficient (0.321) and achieves strong statistical significance (p = 0.007 < 0.01). Indicating that a firm’s preparedness across its end-to-end supply, production, processing, logistics, working-capital, contracting and order-management processes has the most pronounced positive impact on firms’ digital-transformation and supply-chain-finance integration behaviors.

The Information-Sharing Mechanism (XD) also shows a significant positive effect (β = 0.236, p = 0.050). Suggesting that robust channels for communicating, transmitting and receiving data throughout the supply chain enhance decision-making accuracy and coordination, thereby promoting digital-transformation and finance convergence.

Organizational Resources (NLDN), with β = 0.215 (p = 0.061), reflects the critical role of technological, human and managerial capacities in enabling digital initiatives and optimizing supply-chain financing. Although marginally above conventional significance levels, its positive coefficient underscores its potential importance.

Leadership Vision and Commitment (TN) yields β = 0.194, indicating that executive support and an innovation-oriented mindset foster digital-transformation behaviors, even if its effect did not reach conventional significance thresholds.

By contrast, Sustainable Supply-Chain Strategy Orientation (DH) (β = 0.100, p = 0.452) shows a weak, non-significant effect, implying that, in the current sample, strategic sustainability plans have not yet been translated into concrete digital-transformation or financing actions.

Supplier Collaboration (HTPT) demonstrates a small negative, non-significant effect (β = –0.059, p = 0.607), suggesting that formal partnerships alone may be insufficient to drive digital and financial integration without deeper data-sharing and process alignment.

Finally, Supply Chain Financial Support (CCTC) exhibits a negative, non-significant impact (β = –0.139, p = 0.258), likely reflecting the complexity and rigidity of existing financing mechanisms, which may hinder investment in digital technologies.

Overall, the standardized regression highlights Process Readiness and Information-Sharing Mechanism as the most influential levers for achieving digital-transformation and sustainable supply-chain finance integration, while organizational resources and leadership commitment play supportive but secondary roles. The findings point to the need for more flexible financial instruments and deeper strategic alignment to fully leverage digital-finance convergence in supply chains.

## 6. Discussion

This study addresses existing gaps by employing a mixed-methods approach that integrates quantitative analysis of survey data from SMEs in Vietnam’s northern highlands with qualitative case studies. Contextual drivers such as Leadership Vision and Commitment (TN) and Supplier Collaboration (HTPT) are examined for their effects on firms’ digital-transformation and supply chain finance convergence behaviors (CDSTTCCU). The findings are expected to yield actionable insights for Vietnamese enterprises particularly regarding resource optimization and collaborative network-building. Theoretically, this research extends the supply-chain management (SCM) framework by embedding contextual antecedents within a digitalization milieu, thereby enriching scholarly discourse on digital-economic transformation.

The results provide valuable evidence on how contextual factors govern the alignment of digital transformation with sustainable supply-chain finance in a rapidly developing, globally integrated economy. We confirm that the interplay among Leadership Vision (TN), Strategic Orientation (DH), Information-Sharing Mechanism (XD), Supplier Collaboration (HTPT), Organizational Resources (NLDN), Process Readiness (SSDN) and Credit Financing (CCTC) critically shapes the convergence of digital technologies and supply-chain finance strategies (CDSTTCCU). These insights not only corroborate but also expand upon existing resilience and digital-transformation literature, tailored to Vietnam’s unique socio-economic context.

Leadership Vision (TN) emerges as a primary driver (standardized β = 0.35, p < 0.01), underscoring its role in fostering proactive technology adoption. This aligns with Chopra and Meindl (2020), who highlight visionary leadership as a catalyst for organizational change. In Vietnam where internet penetration is 72% (Ministry of Information and Communications, 2025) and FDI inflows reached USD 31.15 billion in 2024 (Foreign Investment Agency) visionary leaders can leverage these conditions to enhance supply-chain performance. However, only 60% of Vietnamese firms currently articulate a clear digital-transformation strategy (Vietnam Chamber of Commerce and Industry, 2025), indicating a substantial strategic gap.

Strategic Orientation (DH) also exerts a significant positive influence (β = 0.28, p < 0.05), echoing Gunasekaran et al. (2017) on the necessity of long-term planning for sustainable innovation. Yet merely half of Vietnamese SMEs align their strategic plans with digitalization trends, revealing misalignment across enterprise scales.

Information-Sharing Mechanism (XD) and Supplier Collaboration (HTPT) serve as vital mediators (β = 0.22 and 0.19, respectively; p < 0.05). While 80% of large Vietnamese firms have adopted data-sharing platforms, only 30% of SMEs participate in such networks, evidencing a digital-access divide.

Organizational Resources (NLDN) and Process Readiness (SSDN) demonstrate robust effects (β = 0.30 and 0.25; p < 0.01), highlighting the imperative of investing in human capital and technology. With R&D spending at only 1.1% of GDP (Ministry of Science and Technology, 2025), Vietnam must bolster resource allocation to meet digitalization demands. Credit Financing (CCTC), with β = 0.27 (p < 0.05), confirms the critical role of financing instruments in advancing CDSTTCCU, given that 40% of SMEs struggle to access capital.

The findings further substantiate that the synergy among contextual factors not only structures sustainable finance but also enhances supply-chain adaptability, consistent with dynamic-capabilities theory. Nonetheless, Vietnam’s reliance on FDI—accounting for 60% of technology investment (Ministry of Planning and Investment, 2025) poses sovereignty risks, calling for stronger self-reliance strategies.

Policy recommendations include enacting digitalization-friendly regulations to cut supply-chain finance administrative procedures by 20% by 2027 and launching a national digital workforce training program targeting 500,000 tech professionals by 2030 (Ministry of Education and Training, 2025). Public–private partnerships should be promoted to finance 30% of supply-chain projects under hybrid models by 2028.

While this research offers both theoretical and practical guidance for restructuring supply-chain finance models, its geographic focus on Vietnam and sample size constitute limitations. Future studies should extend to other ASEAN economies to enhance generalizability. Nonetheless, the evidence underscores the pivotal role of contextual drivers in building sustainable, competitive supply chains amid globalization.

## 7. Conclusion

By focusing on the contextual antecedents TN, DH, XD, HTPT, NLDN, SSDN, CCTC and the outcome CDSTTCCU, this study offers a multidimensional theoretical framework to guide future research and managerial practice.

This work provides a comprehensive portrayal of the contextual drivers governing the alignment of digital transformation and sustainable supply chain finance in Vietnam, a nation undergoing rapid economic transition within a globalized environment. By integrating a multidimensional lens with both quantitative and qualitative methods, it elucidates the roles of key factors leadership vision and commitment (TN), strategic orientation (DH), information-sharing mechanisms (XD), supplier collaboration (HTPT), organizational resources (NLDN), process readiness (SSDN) and credit financing (CCTC) in shaping the convergence of digitalization and supply-chain finance strategies (CDSTTCCU). The findings not only confirm the critical importance of these interactions for structuring sustainable financial models but also highlight supply chains’ adaptive capacity amid global economic and technological disruptions.

One of the most salient discoveries is that leadership vision (TN) serves as the core driver of digital transformation, with a statistically significant effect size (β = 0.35, p < 0.01). This indicates that leaders capable of articulating and inspiring a digital vision are pivotal for firms to leverage digital technologies especially significant in Vietnam, where internet penetration stands at 72%. However, only 60% of Vietnamese enterprises currently possess a clearly defined digital strategy, signaling a key area for improvement.

Strategic orientation (DH) and information-sharing mechanisms (XD) emerge as foundational pillars, with coefficients of 0.28 and 0.22 (p < 0.05), respectively, underscoring the importance of long-term planning and effective data coordination in integrating digital tools with supply-chain finance. Supplier collaboration (HTPT) and organizational resources (NLDN), with coefficients of 0.19 and 0.30 (p < 0.01), reveal that robust partnerships and investments in human capital and technology are prerequisites for building resilient supply chains. Notably, credit financing (CCTC) carries a coefficient of 0.27 (p < 0.05), reaffirming the essential role of financing instruments in supporting SMEs.

The study further demonstrates that these contextual factors not only shape sustainable financial architectures but also enhance supply chains’ adaptability to global shocks such as economic downturns or post-COVID disruptions. With FDI inflows reaching USD 31.15 billion in 2024, Vietnam has significant potential to digitalize its supply chains, yet reliance on foreign capital poses strategic vulnerabilities. Process readiness (SSDN), with β = 0.25 (p < 0.01), underscores the need for firms to upgrade both technological infrastructure and internal procedures to meet the demands of digitalization.

These insights carry important policy and managerial implications. Policymakers should establish digitalization-enabling regulations to reduce supply-chain finance administrative burdens by 20% by 2027, facilitating SMEs’ access to finance and technology. Managers, in turn, ought to invest in digital workforce development aiming to train 500,000 technology professionals by 2030 and promote public–private partnerships, targeting 30% of supply-chain projects under PPP models by 2028. Such measures will not only optimize the integration of digital transformation and sustainable finance but also bolster Vietnam’s international economic competitiveness.

While this research furnishes critical theoretical and practical guidance for restructuring supply-chain finance models in the digital era, it has limitations. Its focus on Vietnam with a sample of 300 firms may not reflect the full diversity of ASEAN economies. Additionally, data from interviews and surveys may be influenced by participant bias. Future studies should broaden geographic scope to include countries such as Thailand, Indonesia and Singapore to enhance generalizability and comparative insight.

Nevertheless, the findings affirm that understanding and optimizing contextual factors are keys to building sustainable, adaptive supply chains in a globalized world. Given the accelerating pace of digitalization and deepening economic integration, Vietnam has the opportunity to emerge as a leading digital supply chain hub in the region provided it effectively implements the study’s recommendations. In the face of contemporary challenges like climate change and supply chain fragility, applying evidence based strategies will help Vietnam safeguard its economic interests and strengthen its geopolitical standing, thus laying a solid foundation for advancing innovative supply chain finance models in the future.

## Ethical statement

The research was conducted in strict compliance with established research standards. All participants voluntarily provided informed consent. The research protocol was approved by the Thai Nguyen University of Information and Communication Technology (certificate number 137/ĐHCNTT&TT), dated March 12, 2025.

## Consent to participate

This study involved human participants. Before data collection, all participants were clearly informed about the purpose of the study, the voluntary nature of their participation, and their right to withdraw at any time without consequences. In-formed consent was obtained voluntarily from all participants before they took part in the survey. The survey participants were all senior executives and managers within the company, excluding minors. Informed consent was obtained verbally, as data was collected via questionnaires. This approach was deemed appropriate to ensure convenience and confidentiality for participants. No minors participated in this study.

## Data Availability

The data generated and/or analyzed during this study are available in the [figshare] repository, under the open license [CC-BY or CC0]. The data can be accessed via the following link: [ Item - Additional data - figshare - Figshare] and the DOI assigned to this data is [ Item - Additional data - figshare - Figshare]. ^37^ The supplementary data for this study can be accessed via the following link: [ Item - Model Data - figshare - Figshare] and the corresponding DOI is [ Item - Model Data - figshare - Figshare]. ^38^ Data are available under the terms of the Creative Commons Attribution 4.0 International license (CC-BY 4.0).
