# Investigating Contextual Factors Affecting Digital Transformation Synergy and Supply Chain Financial Sustainability Models

**DOI:** 10.12688/f1000research.174111.2

**Published:** 2026-05-14

**Authors:** Nguyen Thi Hang, Nam Vu Quynh

**Affiliations:** 1 Thai Nguyen University of Information and Communication Technology, Thai Nguyen, Vietnam; 2 Thai Nguyen University of Economics and Business Administration, Thái Nguyên, Thai Nguyen, Vietnam

**Keywords:** Digital transformation, Supply chain finance, Sustainable financial model, Strategic integration, Supply chain adaptability

## Abstract

**Background:**

In the context of accelerating digital transformation, the alignment between digital transformation initiatives and sustainable supply chain finance has become an important issue for firms seeking to improve operational coordination and financial efficiency. This alignment, however, is influenced not only by technological adoption but also by organizational and contextual conditions such as governance structures, strategic orientation, and resource availability. Existing studies have not sufficiently examined how these contextual factors jointly shape the integration of digital transformation and supply chain finance.

**Methods:**

This study adopts a mixed-methods approach that combines quantitative analysis based on survey data with qualitative insights. A multidimensional analytical framework is developed to examine the contextual factors affecting the integration between digital transformation and sustainable supply chain finance. The framework includes leadership vision, strategic orientation, information-sharing mechanisms, supplier collaboration, organizational resources, process readiness, and access to financial and credit resources.

**Results:**

The findings indicate that these contextual factors collectively influence the degree of alignment between digital transformation initiatives and sustainable supply chain financial models. Leadership vision and clear strategic orientation play a central role in guiding coordination among supply chain actors. Effective information-sharing mechanisms support collaboration and transparency across partners. In addition, organizational resources and process readiness significantly affect the feasibility and effectiveness of implementing digital solutions linked to supply chain finance. Access to financial and credit resources also conditions firms’ ability to operationalize such initiatives.

**Conclusion:**

The study provides empirical evidence on how organizational and contextual conditions shape the integration of digital transformation with sustainable supply chain finance. The results suggest that alignment depends on a combination of strategic direction, internal capabilities, and coordination mechanisms, offering useful implications for firms seeking to design digital transformation initiatives that are consistent with their organizational capacities and operational contexts.

## 1. Introduction

In the context of globalization and the rapid evolution of the digital economy, sychronizing digital transformation (DT) and sustainable financial models in supply chains (SCF) has become an urgent requirement for enhancing enterprise competitiveness and adaptability. The Fourth Industrial Revolution with the emergence of advanced technologies such as artificial intelligence, blockchain and the Internet of Things (IoT), has reshaped traditional supply chain operations, necessitating a shift toward digital ecosystems. ^5^ Simultaneously, the financial sustainability of supply chains including financing mechanisms, credit support and capital optimization has become a key concern amid economic uncertainty and rising operational costs. ^7^ This study focuses on analyzing the contextual factors influencing the synchronization between digital transformation and sustainable supply chain finance models, arguing that this convergence is a core driver for innovation, economic viability and market responsiveness.

The concept of digital transformation in supply chain management refers to the strategic integration of digital technologies to optimize processes, enhance visibility and improve decision-making across the entire supply network. ^11, 12, 14, 28– 35^ Recent studies suggest that digital transformation enables real-time data sharing, predictive analytics and process automation, thereby minimizing waste and enhancing supply chain agility. ^6, 21– 27^ However, the success of digital transformation depends on a set of multidimensional contextual factors, including leadership vision, organizational readiness and inter-organizational collaboration. Likewise, sustainable supply chain finance involves deploying financial tools such as trade finance, supply chain loans and credit guarantees to ensure liquidity and long-term operational stability. The convergence of these two domains is particularly critical in emerging economies, where resource and infrastructure constraints pose significant barriers to adoption. ^1, 36^

In the Vietnamese context, the northern mountainous provinces represent a unique case study, with an emerging entrepreneurial ecosystem and increasing emphasis on sustainable development. This study emphasizes the need to explore how contextual factors shape the integration between digital transformation and supply chain finance, especially in resource scarce regions.

The proposed research model identifies eight key contextual factors influencing this integration: (1) TN – Leadership vision and commitment to implementing digital transformation and supply chain finance; (2) DH – Strategic orientation toward sustainable supply chain management; (3) XD – Information exchange mechanisms within the supply chain; (4) HTPT – Collaborative development of sustainable strategic relationships with suppliers; (5) NLDN – Organizational resources affecting digital transformation and financial optimization; (6) SSDN – Readiness in supply, production, logistics and financial management processes; (7) CCTC – Provision of financial and credit support across the supply chain; (8) CDSTTCCU – Behaviors related to digital transformation and supply chain finance. These elements form a multidimensional framework that illuminates the interaction between technological innovation and financial sustainability.

Existing literature highlights the transformative potential of digital transformation in supply chain management. For example, emphasizes the role of digital tools in improving supply chain visibility, ^13^ while under scores the importance of blockchain in securing financial transactions. ^20^ However, the literature also reveals gaps in understanding how contextual factors mediate this transformation, especially in developing economies. Suggest that leadership commitment and resource availability are critical drivers, yet the specific role of supplier collaboration and process readiness remains under-explored. ^21^ Moreover, the financial aspect of supply chain finance, as discussed by Hofmann et al. (2011), demands a nuanced approach when integrated with digital transformation an area lacking empirical evidence. ^15– 17^

This study addresses these gaps by employing a mixed-methods approach, combining quantitative survey data analysis with qualitative insights from case studies in Northern Vietnam. The study contends that synchronizing digital transformation and supply chain finance is not merely a technical endeavor but a strategic alignment influenced by context-specific economic variables. For instance, limited technological infrastructure in mountainous provinces necessitates innovative financial models to support digital adoption a challenge this research seeks to clarify. By examining the proposed factors, the study aims to provide a comprehensive understanding of how businesses can leverage digital transformation to enhance the sustainability of supply chain finance, thereby contributing to broader discourse on digital economic transformation.

The significance of this study lies in its potential to inform both policy and managerial practice. In Vietnam, where the government has launched a National Digital Transformation Program through 2025 with a vision toward 2030, the findings may guide policymakers in designing incentives to integrate digital transformation and supply chain finance. For businesses, the research offers practical insights into optimizing resource allocation and fostering collaborative networks. Moreover, the academic contribution is substantial, as it expands the theoretical framework of supply chain management by integrating digital transformation and financial sus tainability through a contextual lens an area ripe for deeper scholarly exploration.

## 2. Literature review

The alignment between digital transformation (DT) and sustainable supply chain finance (SCF) has attracted increasing attention as an integrated approach in the context of globalization and Industry 4.0. ^2^ Rather than treating these as two parallel domains, recent studies suggest that meaningful outcomes tend to emerge only when DT and SCF reinforce each other under specific contextual conditions. This perspective calls for a more systematic examination of a set of interrelated factors, including leadership vision (TN), strategic orientation (DH), information sharing mechanisms (XD), supplier collaboration (HTPT), organizational resources (NLDN), process readiness (SSDN), financial and credit support (CCTC), and firms’ behavioral engagement with DT and SCF practices (CDSTTCCU). These dimensions are particularly relevant in the Vietnamese context especially in Northern mountainous regions where resource constraints mean that integration does not occur organically but is highly dependent on organizational and environmental conditions.

In the domain of digital transformation in supply chains, earlier studies largely emphasized the role of technology in operational optimization. Tools such as predictive analytics and blockchain have been shown to enhance visibility, improve automation, and reduce inefficiencies. ^9, 10, 18^ However, a closer reading of this literature suggests that these outcomes cannot be attributed to technology alone. Campos et al., for instance, also highlight the importance of leadership vision (TN) and organizational resources (NLDN) as enabling conditions for technology to deliver value. This argument is reinforced by later work (e.g., Lerman et al., 2022; Kurniawan et al., 2024), which positions leadership commitment as a key lever for digital transformation, particularly in emerging economies where infrastructural limitations persist. ^19, 20^ In this sense, DT is less about the presence of technology per se and more about an organization’s capacity to absorb and effectively deploy it.

From the perspective of supply chain finance, Hofmann et al. (2011) and Caniato et al. (2019) conceptualize SCF as a mechanism through which the credit strength of large firms can be leveraged to improve liquidity for smaller suppliers, especially SMEs. ^8, 17^ Instruments such as receivables financing and invoice discounting have proven useful in easing cash flow constraints. That said, Gelsomino et al. (2016) point out that SCF cannot function effectively in the absence of robust information-sharing mechanisms (XD) and strong inter-firm collaboration (HTPT). ^15^ This reveals a clear point of convergence: effective SCF requires transparent and reliable information, which in turn depends heavily on the level of digital transformation. At the same time, factors such as process readiness (SSDN) and financial support (CCTC) are frequently highlighted as foundational conditions for sustaining financial performance. ^3, 4^

When these two strands of literature are considered together, a common limitation becomes apparent. Much of the existing work examines individual factors in isolation rather than exploring how they interact within a broader system. Variables such as CCTC, DH, SSDN, and CDSTTCCU have been identified, yet their interdependencies remain insufficiently understood. In practice, these elements do not operate independently; they may reinforce or weaken one another. For example, financial support (CCTC) may have limited impact if firms lack adequate process readiness (SSDN), or if strategic orientation (DH) is unclear.

This gap is even more evident in developing economy settings. According to a 2024 report by the IFC and WTO, SMEs in Vietnam account for approximately 40% of GDP and 60% of employment, yet only about 20% have access to formal bank financing. While this underscores the importance of financial support mechanisms (CCTC), it also raises a critical question: why do similar policies yield different outcomes across firms? A plausible explanation lies in variations in strategic orientation, process readiness, and organizational capacity. However, current studies have not sufficiently examined the moderating role of these factors in the DT–SCF relationship.

In addition, empirical evidence on firms’ DT–SCF-related behaviors (CDSTTCCU) remains limited in specific contexts such as Northern mountainous regions of Vietnam. These areas face clear constraints in both digital infrastructure and financial resources, making the processes of digital transformation and supply chain integration more challenging than in urban settings. For instance, while adopting digital platforms such as blockchain may improve financial transparency, the actual benefits depend heavily on process readiness (SSDN) a factor that has not been adequately tested in this context. The lack of empirical data partly explains why policy recommendations often remain general and difficult to operationalize.

Taken together, these observations point to a gap on both theoretical and practical fronts. Theoretically, existing models do not fully capture how contextual factors interact to generate DT–SCF synergy. Practically, this limits the ability to design targeted interventions for SMEs, particularly within national digital transformation initiatives. In response, this study focuses on TN, DH, XD, HTPT, NLDN, SSDN, CCTC, and CDSTTCCU to examine how these factors interact within the Vietnamese context. Drawing on empirical evidence from Northern mountainous regions, the study aims to clarify the mechanisms through which DT and SCF reinforce each other, thereby contributing to both theory development and policy implications for sustainable supply chain practices.

## 3. Research model and hypotheses

In the context of digital transformation (DT) increasingly reshaping supply chain operations, the development of sustainable supply chain finance (SCF) models is no longer purely a matter of financial tools but is closely tied to firms’ internal capabilities. This suggests that the effectiveness of SCF depends on how organizational factors are configured and managed within a digital environment. From this perspective, the research model is developed using an integrative approach, drawing on the resource-based view (RBV) proposed by Wernerfelt (1984), ^31^ building on Penrose (1959), ^27^ and incorporating recent studies on digital transformation in logistics and supply chain finance. This approach helps explain how internal resources and capabilities contribute to competitive advantage, while also clarifying the mechanisms through which DT and SCF may reinforce each other.

The model is designed to identify contextual factors influencing digital transformation and supply chain finance behavior (CDSTTCCU) ( Table 1). Rather than examining these factors in isolation, CDSTTCCU is treated as an aggregated outcome that reflects the extent to which digital technologies are meaningfully integrated into financial and supply chain activities. Accordingly, seven independent variables are included: leadership vision and commitment (TN), strategic orientation (DH), information exchange mechanisms (XD), supplier collaboration (HTPT), organizational resources (NLDN), process readiness (SSDN), and financial and credit support (CCTC). These variables are selected not only based on prior literature but also because they represent core operational conditions within firms.

**Table 1.** Contextual factors influencing the synchronization of digital transformation and SCF.

Factor	Description	References
TN	Vision and commitment of company leadership toward implementing the integration of digital transformation and supply chain finance.	Dharmayanti et al., 2023; Alkaraan et al., 2022
DH	Strategic orientation and deployment of sustainable supply chain management strategies by the enterprise.	Deckert, 2023; Campos et al., 2016; Cherrafi et al., 2018
XD	Establishing effective mechanisms for information exchange, transmission and reception throughout the entire supply chain.	Gelsomino et al., 2016; Guandalini, 2022; Mariadoss, 2011
HTPT	Collaboration and development of sustainable strategic relationships between enterprises and their suppliers.	Hofmann & Belin, 2011; He, 2020; Omrani, 2022
NLDN	Organizational resource factors influencing the implementation of digital transformation and optimization of supply chain finance.	Ali, 2023; Jia, 2025; Nejati, 2017; Neri, 2023; Sullivan et al., 2018
SSDN	The enterprise’s readiness across supply, production, processing, transportation, capital management, contracts and order fulfillment processes, impacting the implementation of digital transformation and the optimization of supply chain finance.	Caniato et al., 2019; Plekhanov et al., 2023; Zhao et al., 2024; Sun et al., 2025
CCTC	Provision of financial resources and credit support across the entire supply, production, processing, transportation, capital management, contracts and order fulfillment processes, influencing the implementation of digital transformation and the optimization of supply chain finance.	Wang et al., 2024; Zangiacomi et al., 2020
CDSTTCCU	Behavior related to the implementation of digital transformation and supply chain finance.	Weritz et al., 2025; Yuan et al., 2024

More specifically, leadership vision and commitment (TN) act as a driving force that initiates and guides transformation efforts, while strategic orientation (DH) translates this vision into concrete supply chain management practices aligned with sustainability goals. Information exchange mechanisms (XD) and supplier collaboration (HTPT) provide the foundation for data connectivity and coordination across the supply chain. At the same time, organizational resources (NLDN) and process readiness (SSDN) reflect the firm’s capacity to absorb and implement digital solutions. Financial and credit support (CCTC), in turn, ensures liquidity and supports financial flows throughout the supply chain. Based on this structure, CDSTTCCU is specified as the dependent variable, capturing the combined effect of these contextual factors.

Building on this logic, the hypotheses are formulated to test the direct effects of each contextual factor on CDSTTCCU. Specifically, TN, DH, XD, HTPT, NLDN, SSDN, and CCTC are all expected to have positive impacts on digital transformation and supply chain finance behavior. This setup allows the study to assess the relative importance of each factor within an integrated framework, thereby shedding light on how the synergy between DT and SCF emerges in practice.

## 4. Research methodology

This study adopts a mixed-method approach to capture both contextual depth and empirical validation. Rather than combining qualitative and quantitative methods in parallel, the research is designed as a sequential explanatory process. In this design, the qualitative phase serves to refine constructs and contextualize the model, while the quantitative phase is used to test the proposed hypotheses.

In the first stage, qualitative data were collected through 25 semi-structured interviews with senior managers from firms and banks in Vietnam. Participants were selected based on their experience with digital transformation and supply chain finance strategies, ensuring that the insights reflect actual managerial practices. The interviews focused on key factors including TN, DH, XD, HTPT, NLDN, SSDN, and CCTC, allowing the study to better understand how these elements are interpreted and applied in practice. Importantly, findings from this stage are not treated as standalone evidence but are used to refine the measurement scales and improve the contextual relevance of the survey instrument.

In the second stage, a structured survey was conducted with 78 firms, yielding 66 valid responses, corresponding to a response rate of 84.6%. The sample includes both small and medium-sized enterprises (SMEs) and large firms, selected using a stratified sampling approach to ensure representation across industries and firm sizes. A five-point Likert scale was employed to measure perceptions of the identified factors and their influence on CDSTTCCU, consistent with prior research in organizational behavior and digital transformation.

For data analysis, multiple linear regression was applied to test the research model. The analysis followed a step-by-step procedure to ensure methodological rigor. First, model fit was assessed using R ^2^ and adjusted R ^2^, indicating the explanatory power of the independent variables. Next, the overall significance of the model was evaluated using the F-test from the ANOVA table, with a significance threshold of p < 0.05. Subsequently, the significance of each independent variable was examined based on their respective p-values, allowing for the identification of statistically meaningful relationships.

To ensure the robustness of the results, multicollinearity was assessed using variance inflation factors (VIF), with a threshold of VIF < 10. In addition, key regression assumptions were examined using diagnostic plots, including histograms of standardized residuals, normal probability (P–P) plots, and scatterplots, to verify normality and linearity conditions. All analyses were conducted using SPSS, ensuring consistency and reliability in data processing.

## 5. Results

### 5.1 Scale reliability testing

The reliability test results indicate a high level of internal consistency across the measurement scales. Cronbach’s Alpha coefficients range from 0.766 to 0.960, all exceeding the commonly accepted threshold of 0.6, suggesting that the scales are sufficiently reliable for subsequent analyses. In addition, all 49 observed variables exhibit corrected item total correlations above 0.3, satisfying the minimum requirement for factor analysis (see Table 2).

**Table 2.** Cronbach’s alpha reliability coefficients.

Measurement scale	Encryption	Cronbach’s alpha	Number of observed variables
**Vision and commitment of company leadership toward implementing the integration of digital transformation and supply chain finance.**	TN	0,958	6
**Strategic orientation and deployment of sustainable supply chain management strategies by the enterprise.**	DH	0,786	6
**Establishing effective mechanisms for information exchange, transmission and reception throughout the entire supply chain.**	XD	0,960	7
**Collaboration and development of sustainable strategic relationships between enterprises and their suppliers.**	HTPT	0,904	7
**Organizational resource factors influencing the implementation of digital transformation and optimization of supply chain finance.**	NLDN	0,953	12
**The enterprise’s readiness across supply, production, processing, transportation, capital management, contracts and order fulfillment processes, impacting the implementation of digital transformation and the optimization of supply chain finance.**	SSDN	0,945	4
**Provision of financial resources and credit support across the entire supply, production, processing, transportation, capital management, contracts and order fulfillment processes, influencing the implementation of digital transformation and the optimization of supply chain finance.**	CCTC	0,887	4
**Behavior related to the implementation of digital transformation and supply chain finance.**	CDSTTCCU	0,766	3

(Source: SPSS analysis results).

Beyond meeting technical thresholds, these results also suggest that the observed items are meaningfully aligned with the underlying constructs they are intended to capture, rather than merely achieving statistical adequacy. More specifically, the scale for “Information-sharing mechanisms” (XD) reports the highest Cronbach’s Alpha (0.960), followed closely by “Leadership vision” (TN) (0.958), indicating a very strong degree of internal consistency among their respective items. By contrast, the construct “Behavioral engagement in digital transformation and supply chain finance” (CDSTTCCU) records the lowest value (0.766), although it still falls within the acceptable range for exploratory research.

This relatively lower consistency can be attributed to the nature of CDSTTCCU as a composite behavioral construct, which is simultaneously influenced by multiple organizational and environmental factors. As a result, the degree of homogeneity among its indicators tends to be lower than that observed in more perceptual or managerial constructs such as TN or XD.

All 49 items have corrected item–total correlations greater than 0.3, indicating that each item contributes positively to its respective scale, with no need for item removal. The number of items per construct varies from 3 (CDSTTCCU) to 12 (NLDN), reflecting differences in the conceptual breadth of the constructs. Notably, although the “Organizational resources” (NLDN) scale includes a relatively large number of items (12), its Cronbach’s Alpha remains high without substantially exceeding the 0.95 threshold. This suggests that the scale does not suffer from redundancy—a common concern when many items are included.

Taken together, these findings provide a solid basis for proceeding with exploratory factor analysis (EFA), while also ensuring that the dataset is not compromised by poorly performing measurement items. Overall, the scales demonstrate satisfactory reliability and internal consistency, thereby supporting subsequent regression analyses.

It is important to note, however, that Cronbach’s Alpha captures internal consistency but does not assess the unidimensionality of the constructs. Therefore, combining reliability testing with factor analysis is necessary to validate the underlying structure of the variables and to strengthen the robustness of the measurement model.

In this sense, the results go beyond merely satisfying technical criteria. The measurement system appears capable of consistently capturing key contextual factors (TN, DH, XD, HTPT, NLDN, SSDN, CCTC) as well as the dependent construct (CDSTTCCU), thereby providing a reliable foundation for testing the study’s hypotheses in subsequent analyses.

### 5.2 Exploratory Factor Analysis (EFA)

*EFA for Independent Variables*

Kaiser-Meyer-Olkin Measure of Sampling Adequacy (KMO) = 0.587.

The results of the factor analysis indicate that the KMO value is 0.587, which exceeds the minimum threshold of 0.5, confirming the suitability of the dataset for factor analysis. The Bartlett’s Test of Sphericity yields a Chi-square value of 3802.120 with a significance level (Sig.) of 0.000, which is less than 0.05. This result suggests that the observed variables are significantly correlated with each other and the factor analysis is statistically meaningful (see Table 3). Consequently, the null hypothesis (H0), stating that the variables are uncorrelated in the population, is rejected. This implies that the correlation matrix is not an identity matrix and the data meets the necessary conditions for factor extraction.

**Table 3.** KMO and Bartlett’s test.

Kaiser-Meyer-Olkin Measure of Sampling Adequacy		.587
Bartlett’s Test of Sphericity	Approx. Chi-Square	3802.120
	df	1035
	Sig.	.000

(Source: SPSS analysis results)

The factor extraction was conducted using Principal Component Analysis (PCA) with Varimax rotation to optimize factor interpretability. The results show that out of the original 46 observed variables, 6 were excluded and the remaining were grouped into 7 factors. The cumulative variance explained is 78.362%, which is significantly higher than the standard threshold of 50%, indicating that the seven extracted components account for 78.362% of the total variance in the dataset.

Additionally, all extracted factors have eigenvalues greater than 1, with the lowest eigenvalue among them being 1.165, thus meeting the standard criterion for factor retention.

**Table 4.** Rotated component matrix using varimax rotation method.

	Component						
	1	2	3	4	5	6	7
NLDN1	.941						
NLDN10	.934						
NLDN3	.932						
NLDN2	.907						
NLDN4	.863						
NLDN6	.841						
NLDN5	.818						
NLDN9	.719						
NLDN7	.710						
NLDN8	.701						
XD2		.968					
XD1		.952					
XD3		.906					
XD4		.876					
XD6		.841					
XD5		.827					
XD7		.783					
TN4			.913				
TN5			.912				
TN3			.864				
TN1			.855				
TN6			.849				
TN2			.784				
HTPT1				.891			
HTPT2				.851			
HTPT3				.826			
HTPT6				.750			
HTPT7				.728			
HTPT5				.720			
HTPT4				.667			
SSDN2					.914		
SSDN4					.873		
SSDN3					.868		
SSDN1					.794		
CCTC4						.842	
CCTC3						.826	
CCTC1						.751	
CCTC2						.724	
DH2							.786
DH1							.693
DH3							.586
DH4							.552

Extraction Method: Principal Component Analysis.

Rotation Method: Varimax with Kaiser Normalization.

All factor loadings are greater than 0.5 and there are no cases in which a variable loads significantly onto more than one factor with similar loading values. This indicates that the extracted factors satisfy both convergent and discriminant validity in the exploratory factor analysis (EFA). Furthermore, there is no cross-loading or confusion between constructs, meaning that items intended to measure one factor do not inadvertently align with another. Therefore, after the factor analysis, all independent constructs remain unchanged none were added or removed.

The rotated component matrix in the EFA confirms the discriminant validity of the constructs, with all factor loadings exceeding 0.5, which is above the commonly accepted threshold (see Table 4). Consequently, all observed variables are retained for subsequent analyses.

*EFA for the Dependent Variable*

The dependent variable, “Digital Transformation and Supply Chain Finance Implementation Behavior” (CDSTTCCU), consists of three observed indicators: CDSTTCCU1, CDSTTCCU2 and CDSTTCCU3.

The results of the KMO and Bartlett’s Test, Total Variance Explained and Rotated Component Matrix are presented in Table 5 below.

**Table 5.** Results of KMO and Bartlett’s test.

Kaiser-Meyer-Olkin Measure of Sampling Adequacy	.656
Bartlett's Test of Sphericity Approx. Chi-Square	54.467
df	3
Sig.	.000

The results of the KMO and Bartlett’s Test indicate that the KMO coefficient reaches 0.656, satisfying the minimum threshold (> 0.6) required for factor analysis ( Table 5). Meanwhile, Bartlett’s Test of Sphericity yields a Chi-Square value of 54.467, with degrees of freedom (df ) = 3 and a significance level (Sig.) = 0.000 < 0.05. This confirms that the correlation matrix among the observed variables is statistically significant and distinct, thereby justifying the continuation of exploratory factor analysis (EFA). These findings demonstrate that the data are suitable and reliable for subsequent analyses within the proposed research model.

The results of the variance analysis indicate that the first factor has an eigenvalue of 2.049, accounting for 68.3% of the total variance surpassing the theoretical threshold of 50% as required by factor analysis principles ( Table 6). Since only one factor exhibits an eigenvalue greater than 1, the model satisfies the condition for principal component extraction, thereby ensuring theoretical generalizability. This confirms that all observed variables converge to measure a single latent construct, consistent with the theoretical framework concerning contextual factors influencing the synchronization of digital transformation and supply chain finance.

**Table 6.** Total variance explained.

Component	Initial Eigenvalues			Extraction sums of squared loadings		
	Total	% of variance	Cumulative %	Total	% of variance	Cumulative %
1	2.049	68.300	68.300	2.049	68.300	68.300
2	.617	20.570	88.870			
3	.334	11.130	100.000			

### 5.3 Analysis of R ^2^ (R Square) and Adjusted R ^2^

R Square represents the proportion of variance in the dependent variable that is explained by the independent variables in the regression model. A higher R Square value implies that the model explains a greater proportion of the variation in the dependent variable. However, R Square can be inflated by the number of independent variables included in the model. Therefore, Adjusted R Square is used to compensate for the potential overestimation, offering a more accurate measure by adjusting for the number of predictors that do not meaningfully contribute to the model. Both R Square and Adjusted R Square range from 0 to 1 ( Table 7).

**Table 7.** Model summary results.

Model summary ^b^					
Model	R	R square	Adjusted R square	Std. Error of the estimate	Durbin-Watson
1	.659 ^a^	.434	.365	.61939	1.734

^a^ Predictors: (Constant), TN, XD, HTPT, NLDN, SSDN, CCTC, DH.

^b^ Dependent Variable: CDSTTCCU.

The model demonstrates a moderate level of statistical significance, with an R ^2^ value of 43.4%. This indicates that approximately 43.4% of the variance in digital transformation and supply chain finance behavior is explained by contextual factors such as enterprise readiness (SSDN), information exchange mechanisms (XD), organizational resources (NLDN), leadership vision (TN) and strategic orientation (DH).

The Adjusted R ^2^ is about 7% lower than the R ^2^, suggesting that some of the independent variables included in the model may not contribute significantly or may introduce noise into the regression. Nonetheless, the gap is not substantial, implying that the model remains statistically reliable and applicable to real world contexts, particularly in the domain of organizational behavior management.

### 5.4 Testing the goodness of fit of the linear regression model

To assess the goodness of fit of the linear regression model with the observed data, it is necessary to conduct hypothesis testing based on the significance value (Sig.) of the F-statistic in the ANOVA table. This test determines whether the overall regression model provides a statistically significant fit to the data ( Table 8).

**Table 8.** ANOVA analysis results.

ANOVA ^a^						
Model		Sum of squares	df	Mean square	F	Sig.
1	Regression	17.040	7	2.434	6.345	.000 ^b^
	Residual	22.252	58	.384		
	Total	39.292	65			

^a^ Dependent Variable: CDSTTCCU.

^b^ Predictors: (Constant), TN, XD, HTPT, NLDN, SSDN, CCTC, DH.

The results indicate that the Total Sum of Squares (Total SS = 39.292) represents the overall variance of the dependent variable (CDSTTCCU). The variance explained by the regression model (Regression SS = 17.040) reflects the portion of variation in the dependent variable accounted for by the independent variables in the model (TN, XD, HTPT, NLDN, SSDN, CCTC, DH). The residual variance (Residual SS = 22.252) represents the portion of variation not explained by the model, attributable to random error.

F = 6.345: This statistic is computed by dividing the Mean Square of the regression by the Mean Square of the residuals. It is used to test the overall significance of the regression model. The corresponding p-value (Sig. = 0.000) is less than the conventional significance level of 0.05, leading to the rejection of the null hypothesis (H _0_) and acceptance of the alternative hypothesis (H _1_). This means that the regression model is statistically significant and fits the data.

Based on the ANOVA table, the significance level of the F-test is 0.000 < 0.05, confirming that the regression model is appropriate for explaining the relationship between the contextual factors and the dependent variable.

The regression model is statistically significant at the 5% level, indicating that at least one independent variable has a significant effect on the dependent variable, CDSTTCCU (Digital Transformation and Supply Chain Finance Implementation Behavior). This validates the model’s applicability for explaining or predicting behavioral outcomes based on the examined contextual factors.

The regression analysis further reveals that the model provides a moderate explanatory power. However, variables that lack statistical significance (such as HTPT and CCTC) may be excluded from the model through coefficient testing or stepwise regression techniques. Additionally, it is recommended to assess multicollinearity (using VIF) and check for homoscedasticity (constant variance of errors) to ensure the model’s robustness.

### 5.5 Multicollinearity diagnostics

To examine the presence of multicollinearity, the Variance Inflation Factor (VIF) was employed. Conventionally, a VIF value greater than 10 indicates significant multicollinearity among independent variables. However, for studies using 5-point Likert scales, researchers have recommended adopting a more conservative threshold of 2. Accordingly, if a variable exhibits a VIF of 2 or higher, multicollinearity is considered to exist. In such cases, the variable in question may not provide meaningful explanatory power regarding the variation of the dependent variable ( Table 9).

**Table 9.** Multicollinearity test results.

Coefficients ^a^								
Model		Unstandardized coefficients		Standardized coefficients	t	Sig.	Collinearity statistics	
		B	Std. Error	Beta			Tolerance	VIF
1	(Constant)	-.169	.753		-.225	.823		
	CCTC	-.163	.143	-.139	-1.142	.258	.659	1.518
	SSDN	.342	.123	.321	2.788	.007	.737	1.357
	NLDN	.252	.132	.215	1.910	.061	.769	1.301
	HTPT	-.064	.123	-.059	-.517	.607	.748	1.338
	XD	.278	.139	.236	1.998	.050	.700	1.428
	DH	.136	.180	.100	.757	.452	.564	1.772
	TN	.224	.135	.194	1.657	.103	.711	1.407

^a^ Dependent Variable: CDSTTCCU.

The regression analysis identified two contextual factors with statistically significant positive effects on the convergence of digital transformation and sustainable supply-chain finance:

Process Readiness(SSDN):β=0.342,p=0.007.

This was the strongest predictor, indicating that a firm’s readiness across its end-to-end supply, production, logistics, working-capital, contracting and order-management processes is a key driver of digital transformation and supply-chain finance integration.

Information−Sharing Mechanism(XD):β=0.278,p=0.050.

Effective channels for exchanging, transmitting and receiving information throughout the supply chain significantly enhance firms’ digital transformation behaviors.

All variance-inflation factors (VIFs) were below 2, confirming the absence of multicollinearity and indicating that the model is stable and reliable for practical application.

The standardized regression equation is therefore:

$$\text{CDSTTCCU}=0.321^{*}\text{SSDN}+0.236^{*}\mathrm{XD}+0.215^{*}\text{NLDN}+0.194^{*}\mathrm{TN}+0.100^{*}\mathrm{DH}–0.139^{*}\text{CCTC}–0.059^{*}\text{HTPT}$$

The regression results reveal that Process Readiness (SSDN) exhibits the highest standardized β coefficient (0.321) and achieves strong statistical significance (p = 0.007 < 0.01). Indicating that a firm’s preparedness across its end-to-end supply, production, processing, logistics, working-capital, contracting and order-management processes has the most pronounced positive impact on firms’ digital-transformation and supply-chain-finance integration behaviors.

The Information-Sharing Mechanism (XD) also shows a significant positive effect (β = 0.236, p = 0.050). Suggesting that robust channels for communicating, transmitting and receiving data throughout the supply chain enhance decision-making accuracy and coordination, thereby promoting digital-transformation and finance convergence.

Organizational Resources (NLDN), with β = 0.215 (p = 0.061), reflects the critical role of technological, human and managerial capacities in enabling digital initiatives and optimizing supply-chain financing. Although marginally above conventional significance levels, its positive coefficient underscores its potential importance.

Leadership Vision and Commitment (TN) yields β = 0.194, indicating that executive support and an innovation-oriented mindset foster digital-transformation behaviors, even if its effect did not reach conventional significance thresholds.

By contrast, Sustainable Supply-Chain Strategy Orientation (DH) (β = 0.100, p = 0.452) shows a weak, non-significant effect, implying that, in the current sample, strategic sustainability plans have not yet been translated into concrete digital-transformation or financing actions.

Supplier Collaboration (HTPT) demonstrates a small negative, non-significant effect (β = –0.059, p = 0.607), suggesting that formal partnerships alone may be insufficient to drive digital and financial integration without deeper data-sharing and process alignment.

Finally, Supply Chain Financial Support (CCTC) exhibits a negative, non-significant impact (β = –0.139, p = 0.258), likely reflecting the complexity and rigidity of existing financing mechanisms, which may hinder investment in digital technologies.

Overall, the standardized regression highlights Process Readiness and Information-Sharing Mechanism as the most influential levers for achieving digital-transformation and sustainable supply-chain finance integration, while organizational resources and leadership commitment play supportive but secondary roles. The findings point to the need for more flexible financial instruments and deeper strategic alignment to fully leverage digital-finance convergence in supply chains.

## 6. Discussion

This study addresses existing gaps by employing a mixed-methods approach that integrates quantitative analysis of survey data from SMEs in Vietnam’s northern highlands with qualitative case studies. Contextual drivers such as Leadership Vision and Commitment (TN) and Supplier Collaboration (HTPT) are examined for their effects on firms’ digital-transformation and supply chain finance convergence behaviors (CDSTTCCU). The findings are expected to yield actionable insights for Vietnamese enterprises particularly regarding resource optimization and collaborative network-building. Theoretically, this research extends the supply-chain management (SCM) framework by embedding contextual antecedents within a digitalization milieu, thereby enriching scholarly discourse on digital-economic transformation.

The results provide valuable evidence on how contextual factors govern the alignment of digital transformation with sustainable supply-chain finance in a rapidly developing, globally integrated economy. We confirm that the interplay among Leadership Vision (TN), Strategic Orientation (DH), Information-Sharing Mechanism (XD), Supplier Collaboration (HTPT), Organizational Resources (NLDN), Process Readiness (SSDN) and Credit Financing (CCTC) critically shapes the convergence of digital technologies and supply-chain finance strategies (CDSTTCCU). These insights not only corroborate but also expand upon existing resilience and digital-transformation literature, tailored to Vietnam’s unique socio-economic context.

Leadership Vision (TN) emerges as a primary driver (standardized β = 0.35, p < 0.01), underscoring its role in fostering proactive technology adoption. This aligns with Chopra and Meindl (2020), who highlight visionary leadership as a catalyst for organizational change. In Vietnam where internet penetration is 72% (Ministry of Information and Communications, 2025) and FDI inflows reached USD 31.15 billion in 2024 (Foreign Investment Agency) visionary leaders can leverage these conditions to enhance supply-chain performance. However, only 60% of Vietnamese firms currently articulate a clear digital-transformation strategy (Vietnam Chamber of Commerce and Industry, 2025), indicating a substantial strategic gap.

Strategic Orientation (DH) also exerts a significant positive influence (β = 0.28, p < 0.05), echoing Gunasekaran et al. (2017) on the necessity of long-term planning for sustainable innovation. Yet merely half of Vietnamese SMEs align their strategic plans with digitalization trends, revealing misalignment across enterprise scales.

Information-Sharing Mechanism (XD) and Supplier Collaboration (HTPT) serve as vital mediators (β = 0.22 and 0.19, respectively; p < 0.05). While 80% of large Vietnamese firms have adopted data-sharing platforms, only 30% of SMEs participate in such networks, evidencing a digital-access divide.

Organizational Resources (NLDN) and Process Readiness (SSDN) demonstrate robust effects (β = 0.30 and 0.25; p < 0.01), highlighting the imperative of investing in human capital and technology. With R&D spending at only 1.1% of GDP (Ministry of Science and Technology, 2025), Vietnam must bolster resource allocation to meet digitalization demands. Credit Financing (CCTC), with β = 0.27 (p < 0.05), confirms the critical role of financing instruments in advancing CDSTTCCU, given that 40% of SMEs struggle to access capital.

The findings further substantiate that the synergy among contextual factors not only structures sustainable finance but also enhances supply-chain adaptability, consistent with dynamic-capabilities theory. Nonetheless, Vietnam’s reliance on FDI—accounting for 60% of technology investment (Ministry of Planning and Investment, 2025) poses sovereignty risks, calling for stronger self-reliance strategies.

Policy recommendations include enacting digitalization-friendly regulations to cut supply-chain finance administrative procedures by 20% by 2027 and launching a national digital workforce training program targeting 500,000 tech professionals by 2030 (Ministry of Education and Training, 2025). Public–private partnerships should be promoted to finance 30% of supply-chain projects under hybrid models by 2028.

While this research offers both theoretical and practical guidance for restructuring supply-chain finance models, its geographic focus on Vietnam and sample size constitute limitations. Future studies should extend to other ASEAN economies to enhance generalizability. Nonetheless, the evidence underscores the pivotal role of contextual drivers in building sustainable, competitive supply chains amid globalization.

## 7. Conclusion

By focusing on the contextual antecedents TN, DH, XD, HTPT, NLDN, SSDN, CCTC and the outcome CDSTTCCU, this study offers a multidimensional theoretical framework to guide future research and managerial practice.

This work provides a comprehensive portrayal of the contextual drivers governing the alignment of digital transformation and sustainable supply chain finance in Vietnam, a nation undergoing rapid economic transition within a globalized environment. By integrating a multidimensional lens with both quantitative and qualitative methods, it elucidates the roles of key factors leadership vision and commitment (TN), strategic orientation (DH), information-sharing mechanisms (XD), supplier collaboration (HTPT), organizational resources (NLDN), process readiness (SSDN) and credit financing (CCTC) in shaping the convergence of digitalization and supply-chain finance strategies (CDSTTCCU). The findings not only confirm the critical importance of these interactions for structuring sustainable financial models but also highlight supply chains’ adaptive capacity amid global economic and technological disruptions.

One of the most salient discoveries is that leadership vision (TN) serves as the core driver of digital transformation, with a statistically significant effect size (β = 0.35, p < 0.01). This indicates that leaders capable of articulating and inspiring a digital vision are pivotal for firms to leverage digital technologies especially significant in Vietnam, where internet penetration stands at 72%. However, only 60% of Vietnamese enterprises currently possess a clearly defined digital strategy, signaling a key area for improvement.

Strategic orientation (DH) and information-sharing mechanisms (XD) emerge as foundational pillars, with coefficients of 0.28 and 0.22 (p < 0.05), respectively, underscoring the importance of long-term planning and effective data coordination in integrating digital tools with supply-chain finance. Supplier collaboration (HTPT) and organizational resources (NLDN), with coefficients of 0.19 and 0.30 (p < 0.01), reveal that robust partnerships and investments in human capital and technology are prerequisites for building resilient supply chains. Notably, credit financing (CCTC) carries a coefficient of 0.27 (p < 0.05), reaffirming the essential role of financing instruments in supporting SMEs.

The study further demonstrates that these contextual factors not only shape sustainable financial architectures but also enhance supply chains’ adaptability to global shocks such as economic downturns or post-COVID disruptions. With FDI inflows reaching USD 31.15 billion in 2024, Vietnam has significant potential to digitalize its supply chains, yet reliance on foreign capital poses strategic vulnerabilities. Process readiness (SSDN), with β = 0.25 (p < 0.01), underscores the need for firms to upgrade both technological infrastructure and internal procedures to meet the demands of digitalization.

These insights offer important reference points for both policymakers and managers. Drawing on the empirical findings, policymakers may consider introducing regulations that facilitate digitalization, with the aim of reducing administrative burdens in supply chain finance by approximately 20% by 2027. Such efforts could help small and medium-sized enterprises (SMEs) improve their access to financial resources and digital technologies. At the firm level, managers may prioritize investments in digital human capital development for instance, targeting the training of around 500,000 technology-skilled workers by 2030, while also promoting collaborative arrangements such as public private partnerships (PPP), with a view to increasing the share of supply chain projects implemented under PPP models to about 30% by 2028.

While this study provides several theoretical and practical implications for advancing supply chain finance models in the digital era, certain limitations remain. Future research could extend the geographical scope to countries such as Thailand, Indonesia, and Singapore in order to enhance comparability and generalizability.

Overall, the findings highlight the importance of understanding and improving contextual factors associated with CDSTTCCU within the scope of this study. In the context of accelerating digitalization and economic integration, Vietnam holds considerable potential to strengthen its position within digital supply chain networks, provided that appropriate strategies are effectively implemented.

Amid ongoing challenges such as climate change and supply chain disruptions, adopting evidence-based strategies informed by empirical research may assist both firms and policymakers in enhancing resilience. In turn, this could contribute to shaping more sustainable supply chain finance models in the future.

## Ethical statement

The research was conducted in strict compliance with established research standards. All participants voluntarily provided informed consent. The research protocol was approved by the Thai Nguyen University of Information and Communication Technology (certificate number 137/ĐHCNTT&TT), dated March 12, 2025.

## Consent to participate

This study involved human participants. Before data collection, all participants were clearly informed about the purpose of the study, the voluntary nature of their participation, and their right to withdraw at any time without consequences. In-formed consent was obtained voluntarily from all participants before they took part in the survey. The survey participants were all senior executives and managers within the company, excluding minors. Informed consent was obtained verbally, as data was collected via questionnaires. This approach was deemed appropriate to ensure convenience and confidentiality for participants. No minors participated in this study.

## Data Availability

The data generated and/or analyzed during this study are available in the [figshare] repository, under the open license [CC-BY or CC0]. The data can be accessed via the following link: [ Item - Additional data - figshare - Figshare] and the DOI assigned to this data is [ Item - Additional data - figshare - Figshare]. ^37^ The supplementary data for this study can be accessed via the following link: [ Item - Model Data - figshare - Figshare] and the corresponding DOI is [ Item - Model Data - figshare - Figshare]. ^38^ The supplementary data for this study can be accessed via the following link: [ Item - Model Data - figshare - Figshare] and the corresponding DOI is [ Item - Model Data - figshare - Figshare]. ^39^ Data are available under the terms of the Creative Commons Attribution 4.0 International license (CC-BY 4.0).
