# Investigating Contextual Factors Affecting Digital Transformation Synergy and Supply Chain Financial Sustainability Models

**DOI:** 10.12688/f1000research.174111.3

**Published:** 2026-06-20

**Authors:** Nguyen Thi Hang, Nam Vu Quynh

**Affiliations:** 1 Thai Nguyen University of Information and Communication Technology, Thai Nguyen, Vietnam; 2 Thai Nguyen University of Economics and Business Administration, Thái Nguyên, Thai Nguyen, Vietnam

**Keywords:** Digital transformation, Supply chain finance, Sustainable financial model, Strategic integration, Supply chain adaptability

## Abstract

**Background:**

In the context of accelerating digital transformation, the alignment between digital transformation initiatives and sustainable supply chain finance has become an important issue for firms seeking to improve operational coordination and financial efficiency. This alignment, however, is influenced not only by technological adoption but also by organizational and contextual conditions such as governance structures, strategic orientation, and resource availability. Existing studies have not sufficiently examined how these contextual factors jointly shape the integration of digital transformation and supply chain finance.

**Methods:**

This study adopts a mixed-methods approach that combines quantitative analysis based on survey data with qualitative insights. A multidimensional analytical framework is developed to examine the contextual factors affecting the integration between digital transformation and sustainable supply chain finance. The framework includes leadership vision, strategic orientation, information-sharing mechanisms, supplier collaboration, organizational resources, process readiness, and access to financial and credit resources.

**Results:**

The findings indicate that these contextual factors collectively influence the degree of alignment between digital transformation initiatives and sustainable supply chain financial models. Leadership vision and clear strategic orientation play a central role in guiding coordination among supply chain actors. Effective information-sharing mechanisms support collaboration and transparency across partners. In addition, organizational resources and process readiness significantly affect the feasibility and effectiveness of implementing digital solutions linked to supply chain finance. Access to financial and credit resources also conditions firms’ ability to operationalize such initiatives.

**Conclusion:**

The study provides empirical evidence on how organizational and contextual conditions shape the integration of digital transformation with sustainable supply chain finance. The results suggest that alignment depends on a combination of strategic direction, internal capabilities, and coordination mechanisms, offering useful implications for firms seeking to design digital transformation initiatives that are consistent with their organizational capacities and operational contexts.

## 1. Introduction

In the context of globalization and the rapid evolution of the digital economy, sychronizing digital transformation (DT) and sustainable financial models in supply chains (SCF) has become an urgent requirement for enhancing enterprise competitiveness and adaptability. The Fourth Industrial Revolution with the emergence of advanced technologies such as artificial intelligence, blockchain and the Internet of Things (IoT), has reshaped traditional supply chain operations, necessitating a shift toward digital ecosystems. ^5^ Simultaneously, the financial sustainability of supply chains including financing mechanisms, credit support and capital optimization has become a key concern amid economic uncertainty and rising operational costs. ^7^ This study focuses on analyzing the contextual factors influencing the synchronization between digital transformation and sustainable supply chain finance models, arguing that this convergence is a core driver for innovation, economic viability and market responsiveness.

The concept of digital transformation in supply chain management refers to the strategic integration of digital technologies to optimize processes, enhance visibility and improve decision-making across the entire supply network. ^11, 12, 14, 28– 35^ Recent studies suggest that digital transformation enables real-time data sharing, predictive analytics and process automation, thereby minimizing waste and enhancing supply chain agility. ^6, 21– 27^ However, the success of digital transformation depends on a set of multidimensional contextual factors, including leadership vision, organizational readiness and inter-organizational collaboration. Likewise, sustainable supply chain finance involves deploying financial tools such as trade finance, supply chain loans and credit guarantees to ensure liquidity and long-term operational stability. The convergence of these two domains is particularly critical in emerging economies, where resource and infrastructure constraints pose significant barriers to adoption. ^1, 36^

In the Vietnamese context, the northern mountainous provinces represent a unique case study, with an emerging entrepreneurial ecosystem and increasing emphasis on sustainable development. This study emphasizes the need to explore how contextual factors shape the integration between digital transformation and supply chain finance, especially in resource scarce regions.

The proposed research model identifies eight key contextual factors influencing this integration: (1) TN – Leadership vision and commitment to implementing digital transformation and supply chain finance; (2) DH – Strategic orientation toward sustainable supply chain management; (3) XD – Information exchange mechanisms within the supply chain; (4) HTPT – Collaborative development of sustainable strategic relationships with suppliers; (5) NLDN – Organizational resources affecting digital transformation and financial optimization; (6) SSDN – Readiness in supply, production, logistics and financial management processes; (7) CCTC – Provision of financial and credit support across the supply chain; (8) CDSTTCCU – Behaviors related to digital transformation and supply chain finance. These elements form a multidimensional framework that illuminates the interaction between technological innovation and financial sustainability.

Existing studies have highlighted the growing importance of digital transformation in supply chain management. For example, prior research has shown that digital technologies can improve supply chain visibility and operational efficiency, ^13^ while other studies have emphasized the contribution of blockchain technology to enhancing transparency and security in supply chain financial transactions. ^20^ In addition, leadership commitment, organizational resources, and technological capability have been identified as important conditions that support digital transformation and improve supply chain performance. ^21^

Despite these contributions, previous research has generally examined digital transformation and supply chain finance as two separate streams of inquiry. Studies on digital transformation have mainly focused on technological innovation and operational improvement, whereas research on supply chain finance has largely concentrated on financing mechanisms, capital accessibility, and cash flow management. ^15– 17^ As a result, limited attention has been given to understanding how digital transformation and supply chain finance can be integrated and how contextual factors may facilitate alignment between these two domains.

Another issue is that much of the existing empirical evidence has been generated in developed economies where technological and financial infrastructures are relatively mature. In emerging economies such as Vietnam, particularly in regions where firms face resource constraints, there is still limited understanding of how factors such as strategic orientation, information-sharing mechanisms, supplier relationships, organizational readiness, and financial support influence the integration of digital transformation and supply chain finance. Addressing this gap is therefore the primary objective of the present study.

This study addresses the above gaps by adopting a mixed-methods approach that combines quantitative survey data with qualitative evidence collected from firms and related organizations in Northern Vietnam. The study argues that alignment between digital transformation and supply chain finance should not be viewed solely as a technological or financial issue. Rather, it emerges from the interaction of multiple contextual factors associated with strategic orientation, organizational conditions, technological capability, and financial support.

The contribution of this study can be considered from three perspectives. First, it examines digital transformation and supply chain finance as interconnected components of a broader system, rather than treating them as separate research domains as is common in much of the existing literature. Second, the study develops and empirically tests a framework comprising the contextual factors that may influence the alignment between digital transformation and supply chain finance. Third, it provides empirical evidence from Vietnam, an emerging economy where digital transformation has become a national priority, while challenges related to technological infrastructure, organizational resources, and access to finance remain evident. By examining these contextual factors, the study offers additional insight into how digital transformation and supply chain finance can be integrated in practice and provides a foundation for the development of more sustainable supply chain models in the digital economy.

## 2. Literature review

The alignment between digital transformation (DT) and sustainable supply chain finance (SCF) has attracted increasing attention as an integrated approach in the context of globalization and Industry 4.0. ^2^ Rather than treating these as two parallel domains, recent studies suggest that meaningful outcomes tend to emerge only when DT and SCF reinforce each other under specific contextual conditions. This perspective calls for a more systematic examination of a set of interrelated factors, including leadership vision (TN), strategic orientation (DH), information sharing mechanisms (XD), supplier collaboration (HTPT), organizational resources (NLDN), process readiness (SSDN), financial and credit support (CCTC), and firms’ behavioral engagement with DT and SCF practices (CDSTTCCU). These dimensions are particularly relevant in the Vietnamese context especially in Northern mountainous regions where resource constraints mean that integration does not occur organically but is highly dependent on organizational and environmental conditions. To provide a stronger theoretical foundation for examining DT-SCF integration, this study draws upon the Resource-Based View (RBV), Dynamic Capability Theory (DCT), and Information Processing Theory (IPT). RBV suggests that firms achieve superior performance when they effectively mobilize valuable organizational resources and capabilities. Building on this perspective, DCT emphasizes the ability of firms to reconfigure resources and adapt to changing technological and market conditions. Meanwhile, IPT argues that organizations operating in uncertain environments require effective information processing mechanisms to support decision-making and coordination. These theoretical perspectives provide a basis for understanding how leadership vision (TN), strategic orientation (DH), information sharing (XD), supplier collaboration (HTPT), organizational resources (NLDN), process readiness (SSDN), financial and credit support (CCTC), and DT-SCF-related behavior (CDSTTCCU) interact to influence the successful integration of digital transformation and supply chain finance.

In the domain of digital transformation in supply chains, earlier studies largely emphasized the role of technology in improving operational efficiency, visibility, and automation. Tools such as predictive analytics and blockchain have been associated with better coordination and reduced operational inefficiencies. ^9, 10, 18^ However, the benefits of digital transformation cannot be explained solely by technology adoption. From the perspectives of the Resource-Based View and Dynamic Capability Theory, digital transformation outcomes depend on the ability of firms to mobilize organizational resources and continuously adapt their capabilities to environmental changes. Leadership vision (TN) provides strategic direction for digital initiatives, while organizational resources (NLDN) determine a firm's capacity to invest in technology, develop employee competencies, and support implementation processes. Consistent with this view, Campos et al., Lerman et al. (2022), and Kurniawan et al. (2024) highlight leadership commitment and resource availability as critical conditions for successful digital transformation, particularly in emerging economies facing infrastructure constraints. ^19, 20^ Therefore, the impact of digital transformation is shaped not only by technological investments but also by the organizational conditions that enable firms to convert technological resources into operational and financial value.

From the perspective of supply chain finance, Hofmann et al. (2011) and Caniato et al. (2019) describe SCF as a mechanism that enhances liquidity and working capital management across supply chain partners. ^8, 17^ While financial instruments such as receivables financing and invoice discounting can alleviate financing constraints, their effectiveness depends on the availability of reliable information and inter-organizational coordination. According to Information Processing Theory, firms operating in complex supply chain environments require efficient information-sharing mechanisms (XD) to reduce uncertainty and facilitate decision-making. Similarly, supplier collaboration (HTPT) helps build trust and supports information exchange among supply chain members. ^15^ In this context, digital transformation strengthens SCF implementation by improving data availability, transparency, and transaction visibility. Furthermore, process readiness (SSDN) and financial support (CCTC) provide the operational and financial foundations necessary for sustaining DT-SCF integration over time. ^3, 4^

When these two streams of literature are examined together, an important limitation becomes evident. Existing studies have primarily investigated the determinants of digital transformation and supply chain finance separately, with limited attention given to their interrelationships. Although factors such as strategic orientation (DH), financial support (CCTC), process readiness (SSDN), and DT-SCF-related behavior (CDSTTCCU) have been recognized as important drivers, little is known about how these factors interact within an integrated DT-SCF framework. In practice, these contextual factors are unlikely to operate independently. Instead, their effects may reinforce or constrain one another, thereby influencing the extent to which digital transformation and supply chain finance can be successfully aligned. This limitation suggests the need for a more integrated perspective that captures the combined influence of organizational, technological, and financial conditions.

This gap is even more evident in developing economy settings. According to a 2024 report by the IFC and WTO, SMEs in Vietnam account for approximately 40% of GDP and 60% of employment, yet only about 20% have access to formal bank financing. While this underscores the importance of financial support mechanisms (CCTC), it also raises a critical question: why do similar policies yield different outcomes across firms? A plausible explanation lies in variations in strategic orientation, process readiness, and organizational capacity. However, current studies have not sufficiently examined the moderating role of these factors in the DT–SCF relationship.

In addition, empirical evidence on firms’ DT–SCF-related behaviors (CDSTTCCU) remains limited in specific contexts such as Northern mountainous regions of Vietnam. These areas face clear constraints in both digital infrastructure and financial resources, making the processes of digital transformation and supply chain integration more challenging than in urban settings. For instance, while adopting digital platforms such as blockchain may improve financial transparency, the actual benefits depend heavily on process readiness (SSDN) a factor that has not been adequately tested in this context. The lack of empirical data partly explains why policy recommendations often remain general and difficult to operationalize.

The review above highlights important gaps in both theory and empirical evidence. Existing studies have not sufficiently explained how contextual factors jointly shape the alignment between digital transformation and supply chain finance. Moreover, empirical evidence from developing economies remains limited, particularly in regions characterized by resource constraints and uneven digital development. Addressing these gaps, the present study examines the roles of TN, DH, XD, HTPT, NLDN, SSDN, CCTC, and CDSTTCCU within the Vietnamese context. By investigating the interactions among these factors, the study seeks to provide a more comprehensive understanding of the conditions that facilitate DT–SCF integration and contribute to sustainable supply chain development.

## 3. Research model and hypotheses

In the context of digital transformation (DT) increasingly reshaping supply chain operations, the development of sustainable supply chain finance (SCF) models is no longer purely a matter of financial tools but is closely tied to firms’ internal capabilities. This suggests that the effectiveness of SCF depends on how organizational factors are configured and managed within a digital environment. From this perspective, the research model is developed using an integrative approach, drawing on the resource-based view (RBV) proposed by Wernerfelt (1984), ^31^ building on Penrose (1959), ^27^ and incorporating recent studies on digital transformation in logistics and supply chain finance. This approach helps explain how internal resources and capabilities contribute to competitive advantage, while also clarifying the mechanisms through which DT and SCF may reinforce each other.

The model is designed to identify contextual factors influencing digital transformation and supply chain finance behavior (CDSTTCCU) ( Table 1). Rather than examining these factors in isolation, CDSTTCCU is treated as an aggregated outcome that reflects the extent to which digital technologies are meaningfully integrated into financial and supply chain activities. Accordingly, seven independent variables are included: leadership vision and commitment (TN), strategic orientation (DH), information exchange mechanisms (XD), supplier collaboration (HTPT), organizational resources (NLDN), process readiness (SSDN), and financial and credit support (CCTC). These variables are selected not only based on prior literature but also because they represent core operational conditions within firms.

**Table 1.** Contextual factors influencing the synchronization of digital transformation and SCF.

Factor	Description	References
TN	Vision and commitment of company leadership toward implementing the integration of digital transformation and supply chain finance.	Dharmayanti et al., 2023; Alkaraan et al., 2022
DH	Strategic orientation and deployment of sustainable supply chain management strategies by the enterprise.	Deckert, 2023; Campos et al., 2016; Cherrafi et al., 2018
XD	Establishing effective mechanisms for information exchange, transmission and reception throughout the entire supply chain.	Gelsomino et al., 2016; Guandalini, 2022; Mariadoss, 2011
HTPT	Collaboration and development of sustainable strategic relationships between enterprises and their suppliers.	Hofmann & Belin, 2011; He, 2020; Omrani, 2022
NLDN	Organizational resource factors influencing the implementation of digital transformation and optimization of supply chain finance.	Ali, 2023; Jia, 2025; Nejati, 2017; Neri, 2023; Sullivan et al., 2018
SSDN	The enterprise’s readiness across supply, production, processing, transportation, capital management, contracts and order fulfillment processes, impacting the implementation of digital transformation and the optimization of supply chain finance.	Caniato et al., 2019; Plekhanov et al., 2023; Zhao et al., 2024; Sun et al., 2025
CCTC	Provision of financial resources and credit support across the entire supply, production, processing, transportation, capital management, contracts and order fulfillment processes, influencing the implementation of digital transformation and the optimization of supply chain finance.	Wang et al., 2024; Zangiacomi et al., 2020
CDSTTCCU	Behavior related to the implementation of digital transformation and supply chain finance.	Weritz et al., 2025; Yuan et al., 2024

More specifically, leadership vision and commitment (TN) act as a driving force that initiates and guides transformation efforts, while strategic orientation (DH) translates this vision into concrete supply chain management practices aligned with sustainability goals. Information exchange mechanisms (XD) and supplier collaboration (HTPT) provide the foundation for data connectivity and coordination across the supply chain. At the same time, organizational resources (NLDN) and process readiness (SSDN) reflect the firm’s capacity to absorb and implement digital solutions. Financial and credit support (CCTC), in turn, ensures liquidity and supports financial flows throughout the supply chain. Based on this structure, CDSTTCCU is specified as the dependent variable, capturing the combined effect of these contextual factors.

Building on this logic, the hypotheses are formulated to test the direct effects of each contextual factor on CDSTTCCU. Specifically, TN, DH, XD, HTPT, NLDN, SSDN, and CCTC are all expected to have positive impacts on digital transformation and supply chain finance behavior. This setup allows the study to assess the relative importance of each factor within an integrated framework, thereby shedding light on how the synergy between DT and SCF emerges in practice.

## 4. Research methodology

This study adopts a mixed-method approach to capture both contextual depth and empirical validation. Rather than combining qualitative and quantitative methods in parallel, the research is designed as a sequential explanatory process. In this design, the qualitative phase serves to refine constructs and contextualize the model, while the quantitative phase is used to test the proposed hypotheses.

In the first stage, qualitative data were collected through 25 semi-structured interviews with managers and specialists working in firms and financial institutions in Vietnam. Participants were selected purposively based on three criteria: (i) at least five years of professional experience in supply chain management, finance, or digital transformation; (ii) direct involvement in digitalization initiatives or supply chain finance activities; and (iii) willingness to share practical insights regarding organizational practices and challenges. The interviewees consisted of managers from manufacturing and trading firms, representatives of commercial banks, and experts with experience in digital transformation projects. Each interview lasted approximately 45–60 minutes and followed a semi-structured protocol covering TN, DH, XD, HTPT, NLDN, SSDN, and CCTC.

Interview records were transcribed and analyzed using a thematic coding approach. The analysis was conducted in three stages, including open coding to identify recurring concepts, axial coding to group related themes, and selective coding to establish relationships among key constructs. The qualitative findings were used to refine item wording, verify the contextual relevance of the proposed constructs, and identify issues requiring further examination in the quantitative phase. Therefore, the qualitative stage served as an important foundation for questionnaire development and interpretation of the empirical findings.

The questionnaire was developed based on measurement items adapted from prior studies on digital transformation, supply chain finance, and supply chain management. The initial instrument was reviewed by five academics and practitioners with expertise in supply chain and financial management to assess content relevance and clarity. Based on their feedback, several items were revised to improve contextual suitability for Vietnamese firms. A pilot survey involving 15 respondents was subsequently conducted to identify ambiguous wording and improve questionnaire structure before the full-scale survey was implemented. In the second stage, a structured survey was conducted with 78 firms, yielding 66 valid responses after data screening, corresponding to a response rate of 84.6%. The study focused on firms operating in manufacturing, processing, trading, and related sectors. Stratified sampling was employed to improve representation across firm size categories and industries. The sampling frame was divided into two principal strata, namely SMEs and large firms, with respondents selected from each group to ensure diversity in organizational characteristics and digital transformation practices.

The final sample size was considered adequate for multiple regression analysis. Following the recommendation of Tabachnick and Fidell (2019), the minimum sample size for regression can be estimated as N ≥ 50 + 8m, where m represents the number of independent variables. However, previous methodological studies also suggest that smaller samples may provide reliable estimates when effect sizes are moderate and measurement quality is satisfactory. Considering the exploratory nature of this study and the relatively homogeneous characteristics of the surveyed firms, the final sample was deemed sufficient for identifying statistically meaningful relationships among the proposed variables.

Prior to hypothesis testing, reliability and validity assessments were conducted using Cronbach’s Alpha and exploratory factor analysis (EFA) to confirm the suitability of the measurement scales for subsequent regression analysis. For data analysis, multiple linear regression was applied to test the research model. The analysis followed a step-by-step procedure to ensure methodological rigor. First, model fit was assessed using R ^2^ and adjusted R ^2^, indicating the explanatory power of the independent variables. Next, the overall significance of the model was evaluated using the F-test from the ANOVA table, with a significance threshold of p < 0.05. Subsequently, the significance of each independent variable was examined based on their respective p-values, allowing for the identification of statistically meaningful relationships.

To ensure the robustness of the results, multicollinearity was assessed using variance inflation factors (VIF), with a threshold of VIF < 10. In addition, key regression assumptions were examined using diagnostic plots, including histograms of standardized residuals, normal probability (P–P) plots, and scatterplots, to verify normality and linearity conditions. All analyses were conducted using SPSS, ensuring consistency and reliability in data processing.

## 5. Results

### 5.1 Scale reliability testing

The reliability test results indicate a high level of internal consistency across the measurement scales. Cronbach’s Alpha coefficients range from 0.766 to 0.960, all exceeding the commonly accepted threshold of 0.6, suggesting that the scales are sufficiently reliable for subsequent analyses. In addition, all 49 observed variables exhibit corrected item total correlations above 0.3, satisfying the minimum requirement for factor analysis (see Table 2).

**Table 2.** Cronbach’s alpha reliability coefficients.

Measurement scale	Encryption	Cronbach’s alpha	Number of observed variables
**Vision and commitment of company leadership toward implementing the integration of digital transformation and supply chain finance.**	TN	0,958	6
**Strategic orientation and deployment of sustainable supply chain management strategies by the enterprise.**	DH	0,786	6
**Establishing effective mechanisms for information exchange, transmission and reception throughout the entire supply chain.**	XD	0,960	7
**Collaboration and development of sustainable strategic relationships between enterprises and their suppliers.**	HTPT	0,904	7
**Organizational resource factors influencing the implementation of digital transformation and optimization of supply chain finance.**	NLDN	0,953	12
**The enterprise’s readiness across supply, production, processing, transportation, capital management, contracts and order fulfillment processes, impacting the implementation of digital transformation and the optimization of supply chain finance.**	SSDN	0,945	4
**Provision of financial resources and credit support across the entire supply, production, processing, transportation, capital management, contracts and order fulfillment processes, influencing the implementation of digital transformation and the optimization of supply chain finance.**	CCTC	0,887	4
**Behavior related to the implementation of digital transformation and supply chain finance.**	CDSTTCCU	0,766	3

(Source: SPSS analysis results).

Beyond meeting technical thresholds, these results also suggest that the observed items are meaningfully aligned with the underlying constructs they are intended to capture, rather than merely achieving statistical adequacy. More specifically, the scale for “Information-sharing mechanisms” (XD) reports the highest Cronbach’s Alpha (0.960), followed closely by “Leadership vision” (TN) (0.958), indicating a very strong degree of internal consistency among their respective items. By contrast, the construct “Behavioral engagement in digital transformation and supply chain finance” (CDSTTCCU) records the lowest value (0.766), although it still falls within the acceptable range for exploratory research.

This relatively lower consistency can be attributed to the nature of CDSTTCCU as a composite behavioral construct, which is simultaneously influenced by multiple organizational and environmental factors. As a result, the degree of homogeneity among its indicators tends to be lower than that observed in more perceptual or managerial constructs such as TN or XD.

All 49 items have corrected item–total correlations greater than 0.3, indicating that each item contributes positively to its respective scale, with no need for item removal. The number of items per construct varies from 3 (CDSTTCCU) to 12 (NLDN), reflecting differences in the conceptual breadth of the constructs. Notably, although the “Organizational resources” (NLDN) scale includes a relatively large number of items (12), its Cronbach’s Alpha remains high without substantially exceeding the 0.95 threshold. This suggests that the scale does not suffer from redundancy—a common concern when many items are included.

Taken together, these findings provide a solid basis for proceeding with exploratory factor analysis (EFA), while also ensuring that the dataset is not compromised by poorly performing measurement items. Overall, the scales demonstrate satisfactory reliability and internal consistency, thereby supporting subsequent regression analyses.

However, Cronbach’s Alpha primarily reflects the internal consistency among observed items and, by itself, does not provide sufficient evidence regarding the validity of the underlying measurement structure. For this reason, reliability assessment was complemented with Exploratory Factor Analysis (EFA) to examine whether the observed variables were grouped in accordance with the proposed theoretical constructs. This approach is consistent with the research design adopted in the present study, which relies on EFA and multiple regression analysis, and has been widely applied in exploratory research on organizational behavior, digital transformation, and supply chain management. Future studies may extend this work by employing structural equation modeling techniques, such as PLS-SEM or CB-SEM, which would allow a more comprehensive assessment of measurement quality through indicators including Composite Reliability (CR), Average Variance Extracted (AVE), and the Heterotrait–Monotrait ratio (HTMT), thereby providing additional evidence for the validity of the measurement model. The EFA results further support the adequacy of the measurement model. The observed variables loaded on their expected factors with acceptable factor loadings and without substantial cross-loading issues. These findings suggest that the measurement items adequately represent their corresponding constructs, including TN, DH, XD, HTPT, NLDN, SSDN, CCTC, and CDSTTCCU. Taken together, the reliability and factor analysis results provide a sufficient basis for the subsequent regression analyses and hypothesis testing procedures.

In this sense, the results go beyond merely satisfying technical criteria. The measurement system appears capable of consistently capturing key contextual factors (TN, DH, XD, HTPT, NLDN, SSDN, CCTC) as well as the dependent construct (CDSTTCCU), thereby providing a reliable foundation for testing the study’s hypotheses in subsequent analyses.

### 5.2 Exploratory Factor Analysis (EFA)

*EFA for Independent Variables*

Kaiser-Meyer-Olkin Measure of Sampling Adequacy (KMO) = 0.587.

Although the KMO value is modest, it exceeds the minimum acceptable threshold of 0.50 suggested by Kaiser (1974), indicating that the sample remains adequate for exploratory factor analysis. The Bartlett’s Test of Sphericity yields a Chi-square value of 3802.120 with a significance level (Sig.) of 0.000, which is less than 0.05. This result suggests that the observed variables are significantly correlated with each other and the factor analysis is statistically meaningful (see Table 3). Consequently, the null hypothesis (H0), stating that the variables are uncorrelated in the population, is rejected. This implies that the correlation matrix is not an identity matrix and the data meets the necessary conditions for factor extraction.

**Table 3.** KMO and Bartlett’s test.

Kaiser-Meyer-Olkin Measure of Sampling Adequacy		.587
Bartlett’s Test of Sphericity	Approx. Chi-Square	3802.120
	df	1035
	Sig.	.000

(Source: SPSS analysis results)

The factor extraction was conducted using Principal Component Analysis (PCA) with Varimax rotation to optimize factor interpretability. The results show that out of the original 46 observed variables, 6 were excluded and the remaining were grouped into 7 factors. The cumulative variance explained is 78.362%, which is significantly higher than the standard threshold of 50%, indicating that the seven extracted components account for 78.362% of the total variance in the dataset.

Additionally, all extracted factors have eigenvalues greater than 1, with the lowest eigenvalue among them being 1.165, thus meeting the standard criterion for factor retention.

**Table 4.** Rotated component matrix using varimax rotation method.

	Component						
	1	2	3	4	5	6	7
NLDN1	.941						
NLDN10	.934						
NLDN3	.932						
NLDN2	.907						
NLDN4	.863						
NLDN6	.841						
NLDN5	.818						
NLDN9	.719						
NLDN7	.710						
NLDN8	.701						
XD2		.968					
XD1		.952					
XD3		.906					
XD4		.876					
XD6		.841					
XD5		.827					
XD7		.783					
TN4			.913				
TN5			.912				
TN3			.864				
TN1			.855				
TN6			.849				
TN2			.784				
HTPT1				.891			
HTPT2				.851			
HTPT3				.826			
HTPT6				.750			
HTPT7				.728			
HTPT5				.720			
HTPT4				.667			
SSDN2					.914		
SSDN4					.873		
SSDN3					.868		
SSDN1					.794		
CCTC4						.842	
CCTC3						.826	
CCTC1						.751	
CCTC2						.724	
DH2							.786
DH1							.693
DH3							.586
DH4							.552

Extraction Method: Principal Component Analysis.

Rotation Method: Varimax with Kaiser Normalization.

All factor loadings are greater than 0.5 and there are no cases in which a variable loads significantly onto more than one factor with similar loading values. This indicates that the extracted factors satisfy both convergent and discriminant validity in the exploratory factor analysis (EFA). Furthermore, there is no cross-loading or confusion between constructs, meaning that items intended to measure one factor do not inadvertently align with another. Therefore, although six observed variables were removed during the extraction process, the underlying theoretical constructs remained unchanged and were retained for subsequent analyses.

The rotated component matrix in the EFA confirms the discriminant validity of the constructs, with all factor loadings exceeding 0.5, which is above the commonly accepted threshold (see Table 4). Consequently, all observed variables are retained for subsequent analyses.

*EFA for the Dependent Variable*

The dependent variable, “Digital Transformation and Supply Chain Finance Implementation Behavior” (CDSTTCCU), consists of three observed indicators: CDSTTCCU1, CDSTTCCU2 and CDSTTCCU3.

The results of the KMO and Bartlett’s Test, Total Variance Explained and Rotated Component Matrix are presented in Table 5 below.

**Table 5.** Results of KMO and Bartlett’s test.

Kaiser-Meyer-Olkin Measure of Sampling Adequacy	.656
Bartlett's Test of Sphericity Approx. Chi-Square	54.467
df	3
Sig.	.000

The results of the KMO and Bartlett’s Test indicate that the KMO coefficient reaches 0.656, satisfying the minimum threshold (> 0.6) required for factor analysis ( Table 5). Meanwhile, Bartlett’s Test of Sphericity yields a Chi-Square value of 54.467, with degrees of freedom (df ) = 3 and a significance level (Sig.) = 0.000 < 0.05. This confirms that the correlation matrix among the observed variables is statistically significant and distinct, thereby justifying the continuation of exploratory factor analysis (EFA). These findings demonstrate that the data are suitable and reliable for subsequent analyses within the proposed research model.

The results of the variance analysis indicate that the first factor has an eigenvalue of 2.049, accounting for 68.3% of the total variance surpassing the theoretical threshold of 50% as required by factor analysis principles ( Table 6). Since only one factor exhibits an eigenvalue greater than 1, the model satisfies the condition for principal component extraction, thereby supporting the theoretical coherence of the construct. This confirms that all observed variables converge to measure a single latent construct, consistent with the theoretical framework concerning contextual factors influencing the synchronization of digital transformation and supply chain finance.

**Table 6.** Total variance explained.

Component	Initial Eigenvalues			Extraction sums of squared loadings		
	Total	% of variance	Cumulative %	Total	% of variance	Cumulative %
1	2.049	68.300	68.300	2.049	68.300	68.300
2	.617	20.570	88.870			
3	.334	11.130	100.000			

### 5.3 Analysis of R ^2^ (R Square) and Adjusted R ^2^

R Square represents the proportion of variance in the dependent variable that is explained by the independent variables in the regression model. A higher R Square value implies that the model explains a greater proportion of the variation in the dependent variable. However, R Square can be inflated by the number of independent variables included in the model. Therefore, Adjusted R Square is used to compensate for the potential overestimation, offering a more accurate measure by adjusting for the number of predictors that do not meaningfully contribute to the model. Both R Square and Adjusted R Square range from 0 to 1 ( Table 7).

**Table 7.** Model summary results.

Model summary ^b^					
Model	R	R square	Adjusted R square	Std. Error of the estimate	Durbin-Watson
1	.659 ^a^	.434	.365	.61939	1.734

^a^ Predictors: (Constant), TN, XD, HTPT, NLDN, SSDN, CCTC, DH.

^b^ Dependent Variable: CDSTTCCU.

The model demonstrates a moderate level of statistical significance, with an R ^2^ value of 43.4%. This indicates that approximately 43.4% of the variance in digital transformation and supply chain finance behavior is explained by contextual factors such as enterprise readiness (SSDN), information exchange mechanisms (XD), organizational resources (NLDN), leadership vision (TN) and strategic orientation (DH).

The Adjusted R ^2^ is about 7% lower than the R ^2^, suggesting that some of the independent variables included in the model may not contribute significantly or may introduce noise into the regression. Nonetheless, the gap is not substantial, implying that the model remains statistically reliable and applicable to real world contexts, particularly in the domain of organizational behavior management.

### 5.4 Testing the goodness of fit of the linear regression model

To assess the goodness of fit of the linear regression model with the observed data, it is necessary to conduct hypothesis testing based on the significance value (Sig.) of the F-statistic in the ANOVA table. This test determines whether the overall regression model provides a statistically significant fit to the data ( Table 8).

**Table 8.** ANOVA analysis results.

ANOVA ^a^						
Model		Sum of squares	df	Mean square	F	Sig.
1	Regression	17.040	7	2.434	6.345	.000 ^b^
	Residual	22.252	58	.384		
	Total	39.292	65			

^a^ Dependent Variable: CDSTTCCU.

^b^ Predictors: (Constant), TN, XD, HTPT, NLDN, SSDN, CCTC, DH.

The results indicate that the Total Sum of Squares (Total SS = 39.292) represents the overall variance of the dependent variable (CDSTTCCU). The variance explained by the regression model (Regression SS = 17.040) reflects the portion of variation in the dependent variable accounted for by the independent variables in the model (TN, XD, HTPT, NLDN, SSDN, CCTC, DH). The residual variance (Residual SS = 22.252) represents the portion of variation not explained by the model, attributable to random error.

F = 6.345: This statistic is computed by dividing the Mean Square of the regression by the Mean Square of the residuals. It is used to test the overall significance of the regression model. The corresponding p-value (Sig. = 0.000) is less than the conventional significance level of 0.05, leading to the rejection of the null hypothesis (H _0_) and acceptance of the alternative hypothesis (H _1_). This means that the regression model is statistically significant and fits the data.

Based on the ANOVA table, the significance level of the F-test is 0.000 < 0.05, confirming that the regression model is appropriate for explaining the relationship between the contextual factors and the dependent variable.

The regression model is statistically significant at the 5% level, indicating that at least one independent variable has a significant effect on the dependent variable, CDSTTCCU (Digital Transformation and Supply Chain Finance Implementation Behavior). This validates the model’s applicability for explaining or predicting behavioral outcomes based on the examined contextual factors.

The regression analysis further reveals that the model provides a moderate explanatory power. While the overall model is statistically significant, only some contextual factors demonstrate statistically significant effects on CDSTTCCU. Therefore, the subsequent coefficient analysis is necessary to identify which factors contribute meaningfully to the model and to distinguish them from variables whose effects are not statistically supported within the present sample. Additionally, it is recommended to assess multicollinearity (using VIF) and check for homoscedasticity (constant variance of errors) to ensure the model’s robustness.

### 5.5 Multicollinearity diagnostics

To examine the presence of multicollinearity, the Variance Inflation Factor (VIF) was employed. Conventionally, a VIF value greater than 10 indicates significant multicollinearity among independent variables. However, for studies using 5-point Likert scales, researchers have recommended adopting a more conservative threshold of 2. Accordingly, if a variable exhibits a VIF of 2 or higher, multicollinearity is considered to exist. In such cases, the variable in question may not provide meaningful explanatory power regarding the variation of the dependent variable ( Table 9).

**Table 9.** Multicollinearity test results.

Coefficients ^a^								
Model		Unstandardized coefficients		Standardized coefficients	t	Sig.	Collinearity statistics	
		B	Std. Error	Beta			Tolerance	VIF
1	(Constant)	-.169	.753		-.225	.823		
	CCTC	-.163	.143	-.139	-1.142	.258	.659	1.518
	SSDN	.342	.123	.321	2.788	.007	.737	1.357
	NLDN	.252	.132	.215	1.910	.061	.769	1.301
	HTPT	-.064	.123	-.059	-.517	.607	.748	1.338
	XD	.278	.139	.236	1.998	.050	.700	1.428
	DH	.136	.180	.100	.757	.452	.564	1.772
	TN	.224	.135	.194	1.657	.103	.711	1.407

^a^ Dependent Variable: CDSTTCCU.

The regression analysis identified two contextual factors with statistically significant positive effects on the convergence of digital transformation and sustainable supply-chain finance:

Process Readiness(SSDN):β=0.321,p=0.007.

This was the strongest predictor, indicating that a firm’s readiness across its end-to-end supply, production, logistics, working-capital, contracting and order-management processes is a key driver of digital transformation and supply-chain finance integration.

Information−Sharing Mechanism(XD):β=0.236,p=0.050.

Effective channels for exchanging, transmitting and receiving information throughout the supply chain significantly enhance firms’ digital transformation behaviors.

All variance-inflation factors (VIFs) were below 2, confirming the absence of multicollinearity and indicating that the model is stable and reliable for practical application.

The standardized regression equation is therefore:

$$\text{CDSTTCCU}=0.321^{*}\text{SSDN}+0.236^{*}\mathrm{XD}+0.215^{*}\text{NLDN}+0.194^{*}\mathrm{TN}+0.100^{*}\mathrm{DH}–0.139^{*}\text{CCTC}–0.059^{*}\text{HTPT}$$

The equation presents the standardized coefficients estimated from the full regression model. However, only SSDN and XD exhibit statistically significant effects at the 5% significance level and therefore constitute the primary explanatory factors in the model.

The regression results reveal that Process Readiness (SSDN) exhibits the highest standardized β coefficient (0.321) and achieves strong statistical significance (p = 0.007 < 0.01). Indicating that a firm’s preparedness across its end-to-end supply, production, processing, logistics, working-capital, contracting and order-management processes has the most pronounced positive impact on firms’ digital-transformation and supply-chain-finance integration behaviors.

The Information-Sharing Mechanism (XD) also shows a significant positive effect (β = 0.236, p = 0.050). Suggesting that robust channels for communicating, transmitting and receiving data throughout the supply chain enhance decision-making accuracy and coordination, thereby promoting digital-transformation and finance convergence.

Organizational Resources (NLDN), with β = 0.215 (p = 0.061), shows a positive association with CDSTTCCU, although the relationship does not reach the conventional level of statistical significance. The result nevertheless suggests that technological, human and managerial capacities may contribute to DT-SCF integration and warrant further investigation in future studies.

Leadership Vision and Commitment (TN) yields a positive coefficient (β = 0.194), but the relationship is not statistically significant (p = 0.103). While leadership may remain an important organizational condition, the present findings do not provide sufficient evidence to confirm a direct effect on CDSTTCCU.

By contrast, Sustainable Supply-Chain Strategy Orientation (DH) (β = 0.100, p = 0.452) shows a weak, non-significant effect, implying that, in the current sample, strategic sustainability plans have not yet been translated into concrete digital-transformation or financing actions.

Supplier Collaboration (HTPT) demonstrates a small negative, non-significant effect (β = –0.059, p = 0.607), suggesting that formal partnerships alone may be insufficient to drive digital and financial integration without deeper data-sharing and process alignment.

Finally, Supply Chain Financial Support (CCTC) exhibits a negative, non-significant impact (β = –0.139, p = 0.258), likely reflecting the complexity and rigidity of existing financing mechanisms, which may hinder investment in digital technologies.

Overall, the regression results identify Process Readiness (SSDN) and Information-Sharing Mechanism (XD) as the only contextual factors exhibiting statistically significant effects on CDSTTCCU. The remaining factors display either weak or statistically insignificant relationships within the present sample, suggesting that their influence may be indirect, context-dependent, or mediated through other organizational capabilities.

## 6. Discussion

This study addresses existing gaps by employing a mixed-methods approach that integrates quantitative analysis of survey data from SMEs in Vietnam’s northern highlands with qualitative case studies. The findings are expected to yield actionable insights for Vietnamese enterprises particularly regarding resource optimization and collaborative network-building. Theoretically, this research extends the supply-chain management (SCM) framework by embedding contextual antecedents within a digitalization milieu, thereby enriching scholarly discourse on digital-economic transformation. The results provide valuable evidence on how contextual factors govern the alignment of digital transformation with sustainable supply-chain finance in a rapidly developing, globally integrated economy. These insights not only corroborate but also expand upon existing resilience and digital-transformation literature, tailored to Vietnam’s unique socio-economic context.

The regression results indicate that only two contextual factors, namely Process Readiness (SSDN) and Information-Sharing Mechanism (XD), exhibit statistically significant effects on Digital Transformation and Supply Chain Finance Behavior (CDSTTCCU). This finding suggests that the successful alignment between digital transformation and supply chain finance depends less on strategic intentions alone and more on firms’ operational preparedness and ability to exchange information effectively across supply chain partners.

Among all examined factors, Process Readiness (SSDN) demonstrates the strongest positive effect on CDSTTCCU (β = 0.321, p = 0.007). This result highlights the importance of having well-established operational procedures, integrated workflows, and organizational preparedness before firms can effectively implement digital transformation initiatives and supply chain finance practices. The finding is consistent with prior studies emphasizing organizational readiness as a prerequisite for successful digital transformation and technological adoption. Firms with greater readiness in procurement, production, logistics, and financial management processes appear to be better positioned to leverage digital technologies and integrate financial solutions throughout the supply chain. In the context of Vietnam, where many firms are still at an early stage of digitalization, process readiness may represent a more immediate determinant of implementation success than broader strategic ambitions.

Information-Sharing Mechanism (XD) also exhibits a positive and statistically significant relationship with CDSTTCCU (β = 0.236, p = 0.050). This finding supports previous research suggesting that transparent, timely, and reliable information exchange forms the foundation for both digital transformation and supply chain finance. Effective information sharing reduces information asymmetry among supply chain participants, improves coordination, and enhances the ability of financial institutions to assess transaction risks. The result is particularly relevant for developing economies, where fragmented information systems often limit both supply chain visibility and access to financing. The evidence from this study reinforces the argument that digital transformation creates value not merely through technology adoption but through the establishment of information-sharing capabilities that support collaboration and financial integration.

Although Organizational Resources (NLDN) and Leadership Vision and Commitment (TN) display positive coefficients, their effects do not reach conventional levels of statistical significance. Nevertheless, the direction of these relationships suggests that organizational capabilities and managerial commitment may still play supporting roles in facilitating digital transformation and supply chain finance initiatives. One possible explanation is that their influence may operate indirectly through process readiness, information-sharing practices, or other organizational mechanisms rather than exerting a direct effect on implementation behavior.

Similarly, Strategic Orientation (DH) does not show a statistically significant relationship with CDSTTCCU. This result may indicate that strategic intentions alone are insufficient to generate tangible implementation outcomes unless they are translated into operational capabilities and concrete organizational actions. While many firms may formally recognize the importance of digital transformation and supply chain finance, the actual implementation process appears to depend more heavily on execution-related factors.

The results also reveal that Supplier Collaboration (HTPT) and Financial Support (CCTC) do not significantly influence CDSTTCCU in the current model. For supplier collaboration, this finding may reflect the reality that collaborative relationships do not automatically lead to digital-financial integration unless they are accompanied by effective information-sharing mechanisms and compatible digital infrastructures. Likewise, the non-significant effect of financial support suggests that access to funding alone may not be sufficient to encourage digital transformation if firms lack the operational capabilities required to utilize such resources effectively.

Taken together, these findings contribute to the emerging literature on the integration of digital transformation and supply chain finance by demonstrating that operational and informational capabilities appear to play a more immediate role than strategic or financial considerations. Existing studies have frequently examined digital transformation and supply chain finance as separate research streams. By empirically investigating their interaction within a unified framework, this study helps clarify the mechanisms through which contextual factors influence the convergence of these two domains. In particular, the findings suggest that process readiness and information-sharing capability serve as critical enablers that translate digitalization efforts into sustainable supply chain finance outcomes.

From a practical perspective, the results offer several implications for firms, policymakers, and financial institutions. Enterprises seeking to strengthen digital transformation and supply chain finance integration should prioritize investments in process standardization, workflow integration, and digital information infrastructure. Financial institutions may improve the effectiveness of supply chain finance programs by supporting digital data connectivity and information transparency among supply chain participants. For policymakers, the findings highlight the importance of promoting interoperable digital platforms, data-sharing standards, and digital capability development programs, particularly for SMEs operating in resource-constrained regions.

Policy recommendations include enacting digitalization-friendly regulations to cut supply-chain finance administrative procedures by 20% by 2027 and launching a national digital workforce training program targeting 500,000 tech professionals by 2030 (Ministry of Education and Training, 2025). Public–private partnerships should be promoted to finance 30% of supply-chain projects under hybrid models by 2028.

While this research offers both theoretical and practical guidance for restructuring supply-chain finance models, its geographic focus on Vietnam and sample size constitute limitations. Future studies should extend to other ASEAN economies to enhance generalizability. Nonetheless, the evidence underscores the pivotal role of contextual drivers in building sustainable, competitive supply chains amid globalization.

## 7. Conclusion

By focusing on the contextual antecedents TN, DH, XD, HTPT, NLDN, SSDN, CCTC and the outcome CDSTTCCU, this study offers a multidimensional theoretical framework to guide future research and managerial practice.

This work provides a comprehensive portrayal of the contextual drivers governing the alignment of digital transformation and sustainable supply chain finance in Vietnam, a nation undergoing rapid economic transition within a globalized environment. By integrating a multidimensional lens with both quantitative and qualitative methods, it elucidates the roles of key factors leadership vision and commitment (TN), strategic orientation (DH), information-sharing mechanisms (XD), supplier collaboration (HTPT), organizational resources (NLDN), process readiness (SSDN) and credit financing (CCTC) in shaping the convergence of digitalization and supply-chain finance strategies (CDSTTCCU). The findings provide evidence that contextual factors contribute differently to DT–SCF alignment and help explain how firms can strengthen sustainable supply chain finance practices in resource-constrained environments.

One of the central findings of this study is that not all contextual factors contribute equally to the alignment between digital transformation and supply chain finance. Among the seven factors examined, only Process Readiness (SSDN) and Information-Sharing Mechanism (XD) demonstrate statistically significant effects on CDSTTCCU.

Process Readiness (SSDN) emerges as the strongest predictor (β = 0.321, p = 0.007), indicating that firms with greater preparedness in supply, production, logistics, working-capital management, contracting, and order-processing activities are more likely to achieve successful integration between digital transformation and supply chain finance practices. This finding suggests that digital-finance convergence depends not only on technology adoption but also on the readiness of underlying operational processes.

Information-Sharing Mechanism (XD) also exhibits a positive and statistically significant relationship with CDSTTCCU (β = 0.236, p = 0.050). The result highlights the importance of transparent and timely information exchange across supply chain partners. Effective information-sharing systems reduce information asymmetry, improve coordination, and create favorable conditions for both digital transformation initiatives and supply chain finance implementation.

Several other factors, including Organizational Resources (NLDN), Leadership Vision and Commitment (TN), Strategic Orientation (DH), Supplier Collaboration (HTPT), and Supply Chain Financial Support (CCTC), do not reach conventional levels of statistical significance in the regression model. Nevertheless, their coefficients suggest that these factors may still play supporting roles in facilitating DT–SCF alignment. Their influence may be indirect, contingent upon firm-specific conditions, or mediated through operational capabilities and information-sharing practices. This finding indicates that the successful integration of digital transformation and supply chain finance may depend more heavily on execution capabilities and operational readiness than on strategic intentions alone.

These findings offer several implications for policymakers and business managers. For policymakers, improving digital infrastructure and promoting data interoperability across supply chain actors may create more favorable conditions for DT–SCF integration. Policies that support enterprise digitalization should place greater emphasis on enhancing operational readiness and facilitating information exchange rather than focusing solely on technology adoption.

For business managers, the results suggest that investments in process standardization, workflow digitalization, and information-sharing systems may generate greater benefits than isolated investments in technology. Firms seeking to strengthen supply chain finance capabilities should prioritize building internal readiness and improving data transparency across supply chain networks. Such efforts can enhance coordination among supply chain partners and support more efficient financial decision-making.

While this study provides several theoretical and practical implications for advancing supply chain finance models in the digital era, certain limitations remain. Future research could extend the geographical scope to countries such as Thailand, Indonesia, and Singapore in order to enhance comparability and generalizability.

Overall, the findings highlight the importance of operational readiness and information-sharing capabilities in supporting the alignment between digital transformation and supply chain finance. Within the context of Vietnamese enterprises, particularly those operating under resource constraints, successful DT-SCF integration appears to depend less on strategic aspirations alone and more on the ability to establish effective processes and transparent information flows.

As digitalization continues to reshape supply chain structures, firms that strengthen these foundational capabilities may be better positioned to enhance resilience, improve financial sustainability, and respond to changing market conditions. The study therefore contributes both empirical evidence and practical insights for advancing sustainable supply chain finance in emerging economies.

Amid ongoing challenges such as climate change and supply chain disruptions, adopting evidence-based strategies informed by empirical research may assist both firms and policymakers in enhancing resilience. In turn, this could contribute to shaping more sustainable supply chain finance models in the future.

## Ethical statement

The research was conducted in strict compliance with established research standards. All participants voluntarily provided informed consent. The research protocol was approved by the Thai Nguyen University of Information and Communication Technology (certificate number 137/ĐHCNTT&TT), dated March 12, 2025.

## Consent to participate

This study involved human participants. Before data collection, all participants were clearly informed about the purpose of the study, the voluntary nature of their participation, and their right to withdraw at any time without consequences. In-formed consent was obtained voluntarily from all participants before they took part in the survey. The survey participants were all senior executives and managers within the company, excluding minors. Informed consent was obtained verbally, as data was collected via questionnaires. This approach was deemed appropriate to ensure convenience and confidentiality for participants. No minors participated in this study.

## Data Availability

The data generated and/or analyzed during this study are available in the [figshare] repository, under the open license [CC-BY or CC0]. The data can be accessed via the following link: [ Item - Additional data - figshare - Figshare] and the DOI assigned to this data is [ Item - Additional data - figshare - Figshare]. ^37^ The supplementary data for this study can be accessed via the following link: [ Item - Model Data - figshare - Figshare] and the corresponding DOI is [ Item - Model Data - figshare - Figshare]. ^38^ The supplementary data for this study can be accessed via the following link: [ Item - Model Data - figshare - Figshare] and the corresponding DOI is [ Item - Model Data - figshare - Figshare]. ^39^ Data are available under the terms of the Creative Commons Attribution 4.0 International license (CC-BY 4.0).
