# Supplementary material for: Investigating Contextual Factors Affecting Digital Transformation Synergy and Supply Chain Financial Sustainability Models
Source: F1000Res. 2026 Jun 20;15:242. Originally published 2026 Feb 12. [Version 3] doi: 10.12688/f1000research.174111.3 (PMC13687738; doi:10.12688/f1000research.174111.3)
Supplement: Peer review history

# Reviewer report: Reviewer response for version 3

**DOI:** 10.5256/f1000research.203651.r525922

**Published:** 2026-10-22

**Authors:** Joniarto Parung

**Affiliations:** 1 University of Surabaya, Surabaya, Indonesia

1. **Is the work clearly and accurately presented and does it cite the current literature?**

Partly. The topic is relevant, and the authors include recent references. However, the terms “synergy,” “alignment,” and “implementation behavior” need clearer definitions. These terms should also be used consistently throughout the manuscript.

 The references need to be checked carefully. For example, reference 27 is about relativistic physics and does not support the discussion of Penrose’s contribution to the Resource-Based View.

**2. Is the study design appropriate and does the work have academic merit?**

 The study addresses a relevant topic and has academic merit, particularly in the context of Vietnamese firms. However, the explanation of the mixed-methods design needs to be clearer.

 The authors describe the design as “sequential explanatory.” However, they conducted interviews first to refine the constructs and questionnaire, followed by the quantitative survey. This sequence appears more consistent with an exploratory sequential design. The authors should check the terminology and explain how the interview findings were used to develop the questionnaire.

 The dependent variable also needs more explanation. The authors describe it as digital transformation and supply chain finance “implementation behavior,” but the discussion focuses on integration and synergy between these two areas. Please provide the three measurement items and explain how they measure this integration. This clarification is needed to understand whether the study measures integration between the two areas or the implementation of related practices in general.

**3. Are sufficient details of methods and analysis provided to allow replication by others?**

 Not yet. The authors should provide the complete questionnaire, sources of the measurement items, sampling procedures, exclusion criteria, and scoring methods. These details are needed so that other researchers can repeat the study.

 Please also clarify the statements about item removal in Sections 5.1 and 5.2. The manuscript states that no items needed to be removed, but later mentions that six items were excluded during factor analysis. Please identify these six items and explain why they were removed.

**4. Is the statistical analysis and its interpretation appropriate?**

 Multiple regression is appropriate for examining the proposed direct relationships. However, the authors need to provide a stronger explanation of the sample size.

 In Section 4, the authors refer to the rule *N ≥ 50 + 8m*, where *m* is the number of independent variables. With seven independent variables, the minimum sample size based on this rule is *N ≥ 50 + 8(7) = 106 observations*. However, the study reports only 66 valid responses from 78 firms surveyed.

 This does not necessarily mean that the analysis is invalid. However, the authors should explain why 66 responses are sufficient for their analysis, preferably by providing a statistical power assessment.

 The interpretation of some results also needs more caution. Process readiness is statistically significant ( *p* = .007), while information sharing is at the significance threshold ( *p* = .050). Please report the *p*-value for information sharing with more decimal places and interpret this result carefully.

 The authors should also avoid concluding that the model is robust or suitable for practical prediction only because the overall F-test is significant and the VIF values are low.

**5. Are all the source data available to ensure full reproducibility?**

 The manuscript states that the data are available on Figshare. However, I have not yet confirmed whether the uploaded files contain all the data and information needed to reproduce the reported analyses. When I try to click the link it contains message “ The page page cannot be found”

**6. Are the conclusions adequately supported by the results?**

 Only partly. Process readiness has the clearest statistical support, while the evidence for information sharing is borderline. The possible indirect effects of other factors have not been tested and should be presented as possible explanations rather than confirmed findings.

 The conclusions should focus on the associations found in the study. The reported analysis does not establish effects on financial sustainability or provide sufficient support for the specific numerical policy targets proposed by the authors.

Is the work clearly and accurately presented and does it cite the current literature?

Partly

If applicable, is the statistical analysis and its interpretation appropriate?

Partly

Are all the source data underlying the results available to ensure full reproducibility?

No

Is the study design appropriate and is the work technically sound?

No

Are the conclusions drawn adequately supported by the results?

Partly

Are sufficient details of methods and analysis provided to allow replication by others?

No

Reviewer Expertise:

supply chain, sustainability and smart  supply chain

I confirm that I have read this submission and believe that I have an appropriate level of expertise to confirm that it is of an acceptable scientific standard, however I have significant reservations, as outlined above.

# Reviewer report: Reviewer response for version 3

**DOI:** 10.5256/f1000research.203651.r525915

**Published:** 2026-10-21

**Authors:** Anton Mulyono Azis

**Affiliations:** 1 Telkom University, Bandung, West Java, Indonesia

This manuscript addresses a relevant topic by examining the contextual conditions associated with the integration of digital transformation and supply chain finance in Vietnamese firms. The mixed-method design is potentially useful, and the manuscript provides a reasonably clear conceptual framework linking seven contextual factors to digital transformation and supply chain finance implementation behavior.

 The main empirical result is also clear: process readiness (SSDN) is the strongest significant predictor, while information-sharing (XD) is reported at the borderline significance level. However, several issues in the current manuscript still need attention, particularly the consistency and interpretation of the measurement analysis, the adequacy of the regression sample, and the alignment between the reported results and some statements in the abstract and implications.

 1. Is the work clearly and accurately presented and does it cite the current literature? **Partly**

 The manuscript presents a relevant literature base and provides a reasonable theoretical foundation using RBV, Dynamic Capability Theory, and Information Processing Theory. The research gap and contribution are also stated more explicitly.

 However, there are still some internal inconsistencies. Most importantly, the abstract states that the contextual factors collectively influence the integration of digital transformation and sustainable supply chain finance, while the regression results indicate that only SSDN is clearly significant and XD is reported at p = .050. The abstract should therefore reflect the actual empirical evidence more precisely.

 There is also an inconsistency in the description of the number of factors: the manuscript refers to “eight key contextual factors,” although CDSTTCCU is subsequently treated as the dependent variable and only seven variables are predictors.

 2. Is the study design appropriate and is the work technically sound? **Partly**

 The sequential mixed-method design is appropriate in principle, and the qualitative phase is logically positioned to inform the quantitative instrument. The main concern is the relatively small quantitative sample for the statistical model.

 The manuscript explicitly cites the rule **N ≥ 50 + 8m** for multiple regression. With seven independent variables, this corresponds to a minimum of 106 observations, whereas the analysis is based on only 66 valid responses. The authors subsequently argue that smaller samples may be acceptable under certain conditions, but this does not fully resolve the contradiction with the sample-size criterion they themselves report. The implications of the limited sample for statistical power and the stability of the coefficients should therefore be discussed more carefully.

 A second concern relates to the EFA. The independent-variable analysis uses a large number of items relative to N = 66, while the reported KMO is only 0.587. This does not necessarily invalidate the exploratory analysis, but it warrants more cautious claims about construct validity.

 3. Are sufficient details of methods and analysis provided to allow replication by others? **Partly**

 The study provides useful information on the 25 interviews, questionnaire development, expert review, pilot testing, and survey procedure. However, some details remain difficult to reconcile.

 In particular, the measurement analysis reports **49 observed variables** in the reliability section, comprising the seven predictors and the three-item dependent construct. The EFA section, however, states that there were **46 original observed variables**, of which six were excluded. The reported rotated matrix also does not appear to correspond straightforwardly to these numbers. This should be checked and corrected so that the number of items reported in the reliability analysis, EFA, and subsequent regression is internally consistent.

 The sampling description could also be more precise regarding the actual distribution of respondents between SMEs and large firms and across the industries represented.

 4. If applicable, is the statistical analysis and its interpretation appropriate? **Partly**

 Multiple linear regression is a reasonable technique for the stated research objective. The overall model is statistically significant (F = 6.345, p < .001), with R² = .434 and adjusted R² = .365. However, the interpretation should be more cautious given the sample size relative to the number of predictors. The treatment of XD (β = .236, p = .050) also requires clarification. The manuscript states that p < .05 is the significance criterion, yet p = .050 is subsequently described as statistically significant. If the authors retain a 5% criterion, this result should be described as borderline rather than unequivocally significant.

 The manuscript also states that VIF values below 2 “confirm” that the model is stable and reliable. VIF only addresses multicollinearity; it does not by itself establish overall model reliability or robustness. Similarly, the reported diagnostic procedures should be distinguished clearly from evidence that the assumptions were actually satisfied.

 5. Are all the source data underlying the results available to ensure full reproducibility? **Partly**

 The manuscript indicates that datasets and supplementary materials have been deposited in Figshare, which is useful for reproducibility. However, the actual Data Availability Statement in the PDF still contains unresolved placeholders such as “[Item - Additional data - figshare - Figshare]” and repeated “Model Data” entries.

 The authors should replace these placeholders with the actual repository links/DOIs and clearly identify which file contains the data used for each major analysis. A short description of the variables, coding, and data structure would also make the repository substantially more useful for independent verification.

 6. Are the conclusions drawn adequately supported by the results? **Partly**

 The central conclusion regarding process readiness is supported by the regression results. The interpretation of information sharing should, however, reflect its borderline p-value.

 Some broader statements go beyond what the reported analysis directly demonstrates. For example, the discussion links the findings to resilience, financial sustainability, and climate-related challenges, although these outcomes are not directly measured in the regression model.

 The policy recommendations are also unusually specific—for example, reducing supply-chain finance administrative procedures by 20% by 2027, training 500,000 technology professionals by 2030, and financing 30% of supply-chain projects through hybrid models. These targets are not derived from the reported analysis. They should either be supported by appropriate policy sources or presented explicitly as external policy proposals rather than empirical implications of this study.

 Major points to address:

1. **Resolve the sample-size issue.** The manuscript should explain the implications of having N = 66 when the stated regression rule N ≥ 50 + 8m implies N ≥ 106 for seven predictors.
2. **Correct the inconsistency in the number of measurement items**, particularly the 49 items reported for reliability, 46 items reported as the starting point for EFA, the six allegedly excluded items, and the items displayed in the rotated matrix.
3. **Reconsider the strength of the EFA claims**, given the relatively small sample and KMO = 0.587. The analysis should not be presented as stronger evidence of construct validity than the data support.
4. **Clarify the interpretation of p = .050 for XD** and distinguish a borderline result from a conventionally significant result.
5. **Ensure consistency between the abstract and the actual regression findings**, particularly regarding which contextual factors are empirically supported.
6. **Replace the unresolved Figshare placeholders** and clearly map the deposited data to the analyses reported in the manuscript.
7. **Moderate causal and broader sustainability/resilience claims** where they are not directly supported by the variables and design.

Overall recommendation

 The manuscript addresses an interesting and relevant research problem and has a potentially useful empirical contribution. The principal findings are understandable, but several methodological and reporting inconsistencies remain important for evaluating the strength of the evidence. I recommend **further revision**, particularly to resolve the sample-size justification, measurement-item inconsistencies, EFA interpretation, and the alignment between the statistical results and the manuscript’s broader claims.

Is the work clearly and accurately presented and does it cite the current literature?

Partly

If applicable, is the statistical analysis and its interpretation appropriate?

Partly

Are all the source data underlying the results available to ensure full reproducibility?

Partly

Is the study design appropriate and is the work technically sound?

Partly

Are the conclusions drawn adequately supported by the results?

Partly

Are sufficient details of methods and analysis provided to allow replication by others?

Partly

Reviewer Expertise:

Operations Management; Supply Chain Management; Performance Management

I confirm that I have read this submission and believe that I have an appropriate level of expertise to confirm that it is of an acceptable scientific standard, however I have significant reservations, as outlined above.

# Reviewer report: Reviewer response for version 3

**DOI:** 10.5256/f1000research.203651.r495345

**Published:** 2026-10-21

**Authors:** Amit Kohli

**Affiliations:** 1 University Canada West, Vancouver, Canada

The manuscript has improved significantly. However, a few minor changes are required:

1. A thematic analysis table should be included, presenting codes, themes, etc.
2. The sample size justification should be elaborated.
3. In the exploratory factor analysis, you should clearly mention the reason for removing a few questions.

Decision: Accepted with a very minor change.

Is the work clearly and accurately presented and does it cite the current literature?

Partly

If applicable, is the statistical analysis and its interpretation appropriate?

Partly

Are all the source data underlying the results available to ensure full reproducibility?

No

Is the study design appropriate and is the work technically sound?

Partly

Are the conclusions drawn adequately supported by the results?

Partly

Are sufficient details of methods and analysis provided to allow replication by others?

No

Reviewer Expertise:

NA

I confirm that I have read this submission and believe that I have an appropriate level of expertise to confirm that it is of an acceptable scientific standard.

# Reviewer report: Reviewer response for version 3

**DOI:** 10.5256/f1000research.203651.r495344

**Published:** 2026-06-24

**Authors:** Benny Hutahayan

**Affiliations:** 1 University of Brawijaya, Malang, East Java, Indonesia

I have carefully reviewed the revised version of the manuscript and the authors’ responses to the previous comments. The revised manuscript has improved in terms of clarity, structure, methodological explanation, and the alignment between the results and conclusions.

Is the work clearly and accurately presented and does it cite the current literature?

Partly

If applicable, is the statistical analysis and its interpretation appropriate?

Yes

Are all the source data underlying the results available to ensure full reproducibility?

Partly

Is the study design appropriate and is the work technically sound?

Partly

Are the conclusions drawn adequately supported by the results?

Yes

Are sufficient details of methods and analysis provided to allow replication by others?

Partly

Reviewer Expertise:

Business Administration, Human Resources Management, Law, and Governance

I confirm that I have read this submission and believe that I have an appropriate level of expertise to confirm that it is of an acceptable scientific standard.

# Reviewer report: Reviewer response for version 2

**DOI:** 10.5256/f1000research.200868.r490048

**Published:** 2026-06-09

**Authors:** Benny Hutahayan

**Affiliations:** 1 University of Brawijaya, Malang, East Java, Indonesia

In the introduction, the research gap needs to be further clarified. The author needs to explain more explicitly what the main limitations of previous research are and how this article addresses those gaps.

 The novelty of the article is not yet sufficiently strong. The author needs to clarify whether the novelty of the research lies in the Vietnamese context, the combination of digital transformation and supply chain finance, the conceptual model, or the empirical testing of contextual factors.

 Literature Review. The literature review remains largely descriptive and does not yet demonstrate a strong theoretical synthesis. The author needs to explain the relationships between variables more systematically, rather than merely listing previous studies. Each construct must be linked to relevant theory

 The hypotheses formulated are quite clear, but the theoretical justification for each hypothesis remains limited. Each hypothesis should be preceded by a stronger conceptual explanation, not merely a definition of the variables.

 Methodology. The qualitative component lacks detail. The author needs to explain the criteria for selecting the 25 informants, the interview procedures, the qualitative data analysis techniques, the coding process, and how the qualitative results were used in developing the instruments or interpreting the findings.

 The authors need to justify the sample size, for example through a power analysis or relevant methodological references.

 The authors mention using stratified random sampling but do not explain the strata used, the population size, or the sample distribution within each stratum.

 The questionnaire development procedure needs to be explained in greater detail, including the sources of the items, the adaptation process, content validation, and instrument pilot testing.

 Results. The reliability report should not rely solely on Cronbach’s alpha but should also include HTMT, composite reliability, or average variance extracted to strengthen construct validity.

 Discussion. The discussion section is not entirely consistent with the regression results. The author identifies several variables as significant, even though they are not significant in the regression table. Please review and revise this section.

 The discussion section also needs to be expanded to address how your findings relate to previous research, then discuss them in context and explain how your study fills a gap and contributes new insights both academically and in practice.

Is the work clearly and accurately presented and does it cite the current literature?

Partly

If applicable, is the statistical analysis and its interpretation appropriate?

Yes

Are all the source data underlying the results available to ensure full reproducibility?

Partly

Is the study design appropriate and is the work technically sound?

Partly

Are the conclusions drawn adequately supported by the results?

Yes

Are sufficient details of methods and analysis provided to allow replication by others?

Partly

Reviewer Expertise:

Business Administration, Human Resources Management, Law, and Governance

I confirm that I have read this submission and believe that I have an appropriate level of expertise to confirm that it is of an acceptable scientific standard, however I have significant reservations, as outlined above.

# Response

**Published:** 2026-06-09

**Authors:** Nguyen Thi Hang

**Affiliations:** ICTU, Vietnam

1. Thank you for this insightful comment. We have revised the Introduction section to more explicitly identify the research gaps and highlight the novelty of the study.

 Specifically, we clarified that prior studies have largely examined digital transformation and supply chain finance separately, with limited attention to their integration and the contextual factors influencing such integration. We also emphasized the lack of empirical evidence from emerging economies, particularly in resource-constrained contexts such as Vietnam.

 Furthermore, we strengthened the statement of novelty by explicitly highlighting three contributions of the study:

 (1) the integrated perspective linking digital transformation and supply chain finance;

 (2) the development and empirical validation of a contextual-factor model consisting of eight determinants; and

 (3) empirical evidence from Vietnam as an emerging economy undergoing rapid digital transformation.

 These revisions have been incorporated into the Introduction section of the revised manuscript.

 2. Thank you for this valuable comment. We have thoroughly revised the literature review and hypothesis development sections. The revised manuscript now synthesizes prior studies around a unified conceptual framework rather than presenting them descriptively. We explicitly discuss the interrelationships among TN, DH, XD, HTPT, NLDN, SSDN, CCTC, and CDSTTCCU, and connect these constructs to established theoretical perspectives such as RBV, Dynamic Capabilities Theory, Supply Chain Integration Theory, and Information Processing Theory. In addition, each hypothesis is now supported by a stronger conceptual rationale explaining the expected causal mechanism, beyond the presentation of definitions and previous empirical findings.

 3. Methodology:

 We sincerely thank the reviewer for these valuable comments regarding the methodology section. In response, we have substantially expanded the description of the research design and data collection procedures. First, additional details have been provided regarding the qualitative phase, including participant selection criteria, interview procedures, thematic coding processes (open, axial, and selective coding), and the role of qualitative findings in refining measurement items and supporting interpretation of quantitative results. Second, we clarified the questionnaire development process by describing the sources of measurement items, expert review procedures, content validation, questionnaire refinement, and pilot testing before the full survey implementation. Third, we expanded the explanation of the stratified sampling approach, including the strata used, respondent selection procedures, and the rationale for ensuring representation across firm categories. Finally, we added a justification for the sample size based on methodological recommendations for regression analysis and clarified the procedures used to assess measurement reliability and validity before hypothesis testing. These revisions improve the transparency, reproducibility, and methodological rigor of the study.

 4. Results:

 Thank you for this valuable observation. We agree that construct validity should be considered alongside internal consistency when evaluating measurement quality. In the revised manuscript, we have expanded the discussion of measurement assessment and clarified that reliability testing was complemented by exploratory factor analysis (EFA) to examine the adequacy of the underlying construct structure. We also added a discussion acknowledging that indicators such as composite reliability (CR), average variance extracted (AVE), and HTMT are frequently employed in structural equation modeling studies. However, given that the present study adopts an exploratory design based on EFA and multiple regression analysis, the combination of Cronbach’s Alpha and EFA was considered appropriate for assessing the quality of the measurement scales. The relevant explanation has been incorporated into Section 5.1 of the revised manuscript.

 5. Discussion:

 We sincerely thank the reviewer for this important observation. Following the suggestion, we carefully reviewed and substantially revised the Discussion section to ensure full consistency with the regression results. Specifically, only Process Readiness (SSDN) and Information-Sharing Mechanism (XD), which were statistically significant in the regression model, are now interpreted as significant determinants of Digital Transformation and Supply Chain Finance Behavior (CDSTTCCU). Variables TN, DH, NLDN, HTPT, and CCTC are no longer discussed as having significant direct effects. Instead, their potential supporting or indirect roles are interpreted more cautiously and in accordance with the empirical evidence.

 In addition, the Discussion section has been expanded to provide a more systematic comparison between the present findings and prior studies. The revised manuscript clarifies areas of consistency and divergence with existing literature and further explains the contextual implications of the results in the Vietnamese setting. We also strengthened the discussion of theoretical contributions by highlighting the role of process readiness and information-sharing capability as key mechanisms linking digital transformation and supply chain finance. Furthermore, practical implications for enterprises, financial institutions, and policymakers have been elaborated to demonstrate how the findings can support the implementation of sustainable digital supply chain initiatives in developing economies.

 We are sincerely grateful to the reviewer for the thoughtful comments and suggestions. Based on these recommendations, the authors conducted a comprehensive review of the analyses, interpretations, and supporting discussions throughout the manuscript to ensure consistency in the presentation of results and alignment with the study’s objectives and conclusions.

# Reviewer report: Reviewer response for version 1

**DOI:** 10.5256/f1000research.191988.r471043

**Published:** 2026-04-16

**Authors:** Amit Kohli

**Affiliations:** 1 University Canada West, Vancouver, Canada

Summary

 Q1) Is the work clearly and accurately presented, and does it cite the current literature?

 Answer 1) Partly: The structure is logical; the quality is reduced due to repetition and weak synthesis.

 Question 2) Is the study design appropriate and is the work technically sound?

 Answer 2) Partly: No doubt the mixed method design is fine, but the limitation in execution reduces the academic strength.

 Question 3) Are sufficient details of methods and analysis provided to allow replication by others?

 Answer 3) No: Key elements such as survey instruments, interview protocols and analysis procedures were found missing.

 Question 4) If applicable, is the statistical analysis and its interpretation appropriate?

 Answer 4) Partly: The techniques used by the authors are fine, but there is a lack of interpretation errors and lack of robustness.

 Question 5) Are all the source data underlying the results available to ensure full reproducibility?

 Answer 5) No: Database found missing.

 Question 6) Are the conclusions drawn adequately supported by the results?

 Answer 6) Partly: Some conclusions are supported, but others rely on overgeneralizations.

 To conclude, although the article has the potential to be of great value and relevancy, there will be considerable revisions needed to meet acceptable academic standards of indexing or scientific value. Future research projects should include more robust research methods, larger more diverse samples, and more extensive analytical data, in order to increase the understanding of DT/SCF integrations. Expanding research in this area of study is necessary to help facilitate continued success of organizations in achieving sustainability and technology-enabled supply chain performance in today's global economy through digital transformation. The authors should limit conclusions to statistically significant results and make a clear distinction between empirical findings and theoretical implications to enhance the credibility and accuracy of the study. The manuscript does not provide raw data, survey responses, or qualitative evidence. In the current academic climate, open data is essential for establishing the validity and credibility of research results. The lack of supporting materials severely limits transparency.

 Recommendation:

- Revise the manuscript to compare and contrast literature rather than simply listing it.
- Ensure consistent use of formatting to enhance readability and credibility.
- Improve the overall coherence and academic tone of the discussion.
- There is no justification for the sample size and no clear explanation of how qualitative findings inform the quantitative results, provide a detailed description of how data was collected, coded, and analyzed to improve transparency and reproducibility.
- Increase the validity and reliability of the analysis.

Is the work clearly and accurately presented and does it cite the current literature?

Partly

If applicable, is the statistical analysis and its interpretation appropriate?

Partly

Are all the source data underlying the results available to ensure full reproducibility?

No

Is the study design appropriate and is the work technically sound?

Partly

Are the conclusions drawn adequately supported by the results?

Partly

Are sufficient details of methods and analysis provided to allow replication by others?

No

Reviewer Expertise:

Digital transformation

I confirm that I have read this submission and believe that I have an appropriate level of expertise to confirm that it is of an acceptable scientific standard, however I have significant reservations, as outlined above.

# Response

**Published:** 2026-05-03

**Authors:** Nguyen Thi Hang

**Affiliations:** ICTU, Vietnam

Dear Dr. Amit Kohli,

 University Canada West, Vancouver, Canada,

 We would like to express our sincere gratitude for your insightful and constructive comments on our manuscript. Your valuable feedback has enabled us to better recognize the limitations of our study and has provided an important foundation for improving its academic rigor, transparency, and overall quality. We have carefully revised the manuscript in response to your comments, as detailed below:

 1. Clarity, structure, and literature review

 We have thoroughly revised the literature review section to enhance coherence, analytical depth, and logical flow. Specifically:

 Redundant content has been reduced, and transitions between paragraphs have been improved.

 The review has been restructured to adopt a more integrative and comparative approach, rather than a descriptive listing of prior studies.

 2. Research design and methodological rigor

 We have clarified and expanded the methodology section to strengthen the rigor and internal consistency of the research design:

 The rationale for adopting a mixed-methods approach has been elaborated, along with the role of each phase (qualitative and quantitative).

 The procedures for qualitative data collection and analysis have been described in greater detail, including coding processes and their role in refining measurement scales.

 The linkage between qualitative findings and the quantitative model has been clarified to ensure methodological consistency.

 3. Replicability

 Comment: Missing survey instruments, interview protocols, and analytical procedures.

 In response, we have provided comprehensive supplementary materials at:

 https://doi.org/10.6084/m9.figshare.31004422

 The full survey questionnaire is now included in the appendix.

 Detailed data analysis procedures have been added, including coding steps, reliability testing (Cronbach’s Alpha, EFA), and regression modeling.

 4. Statistical analysis and interpretation

 We have revised the manuscript to improve the alignment between empirical results and their interpretation:

 Statistical indicators and evaluation criteria (e.g., Cronbach’s Alpha, EFA, regression) are now more clearly explained.

 The connections between analytical results and research hypotheses have been strengthened.

 Interpretations have been refined to adopt a more cautious tone and to avoid overgeneralization beyond the data.

 5. Data availability and transparency

 Comment: Lack of source data and open data.

 We appreciate this important suggestion and have made the relevant materials publicly available at:

 https://doi.org/10.6084/m9.figshare.32147605

 This addition is intended to enhance transparency and support the reproducibility of our findings.

 6. Conclusions and generalizability

 We have revised the conclusion section to improve its validity and precision:

 Only conclusions supported by statistically significant results are retained.

 A clearer distinction is made between empirical findings and theoretical/policy implications.

 A limitations section has been expanded to better define the scope of generalization.

 7. General recommendations

 We have addressed the reviewer’s broader suggestions as follows:

 Formatting and referencing have been standardized to ensure consistency.

 The academic tone and overall coherence of the manuscript have been improved.

 Additional steps have been taken to strengthen the reliability and validity of the measurement scales.

 Once again, we sincerely appreciate your thoughtful and detailed feedback. These revisions have significantly improved the clarity, methodological rigor, and transparency of the manuscript. We hope that the revised version now better meets the academic standards of the journal, and we would be grateful for your further guidance.

 Yours sincerely,

 Dr. Nguyễn Thị Hằng

 Vice Dean, Faculty of Economics and Administration

 University of Information and Communication Technology, Vietnam
